# Comparative Proteome Profiling of Saliva Between Estrus and Non-Estrus Stages by Employing Label-Free Quantitation (LFQ) and Tandem Mass Tag (TMT)-LC-MS/MS Analysis: An Approach for Estrus Biomarker Identification in *Bubalus bubalis*

**DOI:** 10.3389/fgene.2022.867909

**Published:** 2022-05-13

**Authors:** Laishram Kipjen Singh, Mamta Pandey, Rubina Kumari Baithalu, Abhijeet Fernandes, Syed Azmal Ali, Latika Jaiswal, Suryaprakash Pannu, Neeraj, Tushar K. Mohanty, A. Kumaresan, Tirtha K. Datta, Sudarshan Kumar, Ashok K. Mohanty

**Affiliations:** ^1^ Animal Reproduction, Gynaecology and Obstetrics, ICAR-National Dairy Research Institute, Karnal, India; ^2^ Molecular Reproduction Lab, Animal Biotechnology Center, ICAR-National Dairy Research Institute, Karnal, India; ^3^ Proteomics and Cell Biology Lab, Animal Biotechnology Center, ICAR-National Dairy Research Institute, Karnal, India; ^4^ Division of Proteomics of Stem Cells and Cancer, German Cancer Research Center (DKFZ), Heidelberg, Germany; ^5^ Animal Biotechnology Centre, ICAR-National Dairy Research Institute, Karnal, India

**Keywords:** biomarker, estrus, label-free quantitation, LC-MS/MS, tandem mass tag, saliva

## Abstract

Accurate determination of estrus is essentially required for efficient reproduction management of farm animals. Buffalo is a shy breeder and does not manifest overt signs of estrus that make estrus detection difficult resulting in a poor conception rate. Therefore, identifying estrus biomarkers in easily accessible biofluid such as saliva is of utmost interest. In the current study, we generated saliva proteome profiles during proestrus (PE), estrus (E), metestrus (ME), and diestrus (DE) stages of the buffalo estrous cycle using both label-free quantitation (LFQ) and labeled (TMT) quantitation and mass spectrometry analysis. A total of 520 proteins were identified as DEPs in LFQ; among these, 59 and four proteins were upregulated (FC ≥ 1.5) and downregulated (FC ≤ 0.5) during E vs. PE, ME, and DE comparisons, respectively. Similarly, TMT-LC-MS/MS analysis identified 369 DEPs; among these, 74 and 73 proteins were upregulated and downregulated during E vs. PE, ME, and DE stages, respectively. Functional annotations of GO terms showed enrichment of glycolysis, pyruvate metabolism, endopeptidase inhibitor activity, salivary secretion, innate immune response, calcium ion binding, oocyte meiosis, and estrogen signaling. Over-expression of SERPINB1, HSPA1A, VMO1, SDF4, LCN1, OBP, and ENO3 proteins during estrus was further confirmed by Western blotting. This is the first comprehensive report on differential proteome analysis of buffalo saliva between estrus and non-estrus stages. This study generated an important panel of candidate proteins that may be considered buffalo estrus biomarkers which can be applied in the development of a diagnostic kit for estrus detection in buffalo.

## 1 Introduction

Saliva is an important diagnostic body fluid since it contains explicit biomolecules such as metabolites, hormones, mRNA, miRNA, DNA, and proteins that may be a good source of biomarkers of health and disease (Levine, 1993). Saliva consists of secretion from serous and mucous acinar cells of salivary glands and a small portion originating from the blood (Edgar, 1992; Pedersen et al., 2002). Dietary and physiological variations bring changes in the salivary composition (Prout and Hopps., 1970; Mass et al., 2002). Furthermore, its stable nature, high biological half-life, and non-invasive approach make it preferable to other body fluids for biomarker discovery (Li et al., 2004; Archunan et al., 2014; Shashikumar et al., 2017). Proteomics using a mass spectrometer enables the identification of new biomarker proteins in a variety of bodily fluids, including saliva (Almeida et al., 2021). In recent past years, salivary proteomics has led to the discovery of biomarkers for several disease conditions such as oral squamous cell carcinoma (OSCC) (Jou et al., 2014; Wang et al., 2015), autoimmune disease (Castagnola et al., 2017), and genetic diseases such as Down’s syndrome (Cabras et al., 2013) in humans. Unlike human saliva, the characterization of salivary proteins of farm animals has been little studied. A global proteome analysis of bovine saliva was carried out by Ang et al. (2011) using both non-targeted and targeted proteomics techniques to identify only extracellular proteins. They identified 402 proteins and 45 N-linked glycoproteins in bovine saliva with enrichment of low-abundance proteins.

Buffalo (*Bubalus bubalis*) is a premier livestock species that contributes immensely to milk and meat production (20th Livestock Census, 2019). Nevertheless, specific inherent reproductive problems such as delayed puberty, silent or poor expression of estrus signs, and seasonal anestrus compromise its reproductive efficiency and production potential. Accurate and efficient identification of estrus is essential for successful conception and efficient reproduction management of farm animals. However, estrus detection is difficult in buffaloes because of non-manifestation of overt signs of estrus and higher incidences (29%) of silent estrus, especially during the summer season (Singh et al., 2000; Roy and Prakash, 2009). This leads to difficulty in estrus detection, improper insemination time, and conception failure in buffaloes (Sharma et al., 2008; Das and Khan, 2010). In addition, various estrus detection tools, such as teaser bull parading, tail painting, KaMar heat mounting detector, behavioral monitoring using a closed circuit television, and activity monitor using a pedometer, were used in buffaloes that are not very efficient since their sensitivity and specificity vary widely (Selvam and Archunan 2017). Therefore, discovering estrus biomarkers in easily accessible body fluids such as saliva is of utmost importance for developing an easy, reliable, and accurate estrus detection method for buffaloes.

Saliva possesses biomarker potential to detect fertile periods in mammals. Saibaba et al. (2021) reported mass spectrometric analysis of salivary proteome profiles of women during the fertile phase of the menstruation cycle as characterized by mass spectrometry. They identified 16 unique and differentially expressed proteins during the ovulatory phase; among them, cystatin-S offers a biomarker potential. Similarly, Muthukumar et al. (2014a) reported proteome profiling of buffalo saliva during the estrous cycle by employing in-gel digestion followed by LC-MS/MS analysis. They identified 179 proteins collectively during proestrus (PE), estrus (E), and diestrus (DE) stages, and 37 proteins are found exclusively during estrus. Among estrus-specific proteins, β-enolase, toll-like receptor (TLR)-4, clusterin, lactoperoxidase, serotransferrin, TGM3, and UBA6 proteins are the most predominant. Our previous study reported global proteome profiling of buffalo saliva during the estrous cycle (Shashikumar et al., 2018) and identified 275, 371, 304, and 565 proteins with ≥2 peptides during PE, E, ME, and DE stages, respectively. Among these, 62 proteins are identified exclusively during the estrus stage, and heat shock 70-kDa protein 1A, 17-beta-hydroxysteroid dehydrogenase type 1, inhibin beta A chain, and testin proteins are found to be the most predominant during estrus. These two studies concluded that salivary secretion contains specific proteins, and their expression varies according to the estrous cycle stages. However, differential proteome analysis using labeled quantitation {tandem mass tag (TMT)} and label-free quantitation (LFQ) coupled to mass spectrometry for estrus biomarker discovery, which is lacking in buffaloes. Therefore, the aim of the present study was to identify candidate estrus biomarkers in saliva of buffaloes. We hypothesized that identifying differentially expressed proteins (DEPs) associated with estrus could lead to the identification of estrus biomarkers in buffaloes. Thus, for the first time, we identified differentially expressed estrus-associated proteins in buffalo saliva by employing both label-free quantitation (LFQ) and labeled quantitation (TMT) coupled to high-resolution mass spectrometry (LC-MS/MS) and their validation using Western blotting.

## 2 Materials and Methods

### 2.1 Experimental Animals

The study was carried out at the Livestock Research Centre, ICAR-National Dairy Research Institute, Karnal, Haryana. Healthy pluriparous Murrah buffaloes (*n* = 15) of 2–4 parity maintained under iso-managerial conditions were included for the study. The nutrient requirement of the animals was provided as per National Research Council (NRC) standards. After completing the voluntary waiting period, all animals underwent gynecological examination using per-rectal examination and transrectal ultrasonography (Model UST-5820-5). For estrus induction, buffaloes with functional corpus luteum (CL) on the ovary are administered with PGF_2_α (Vetmate; 500 µg I/M). Samples were collected from the next spontaneous estrous cycle, and stages of the estrous cycle were categorized as proestrus (PE; day 2 to 1 before the onset of estrus), estrus (E; day 0), metestrus (ME; day+3), and diestrus (DE; day +10). All the sampling was completed during the breeding season, that is, from November to February. All the experiments conducted in this study were approved by the Institute Animal Ethics Committee (42-IAEC-18-9).

### 2.2 Estrus Detection and Confirmation

Estrus in buffaloes was detected by physical observation of estrus signs. Teaser bull parading was carried out twice a day, that is, daily in the morning 5–6 AM and evening 5–6 PM for exhibition of estrus signs. The following estrus signs were observed: standing to be mounted, sniffing/licking the vulva, chin resting, flehmen’s reaction, mounting on or by other buffaloes, restlessness, bellowing, and frequent micturition. The onset of estrus is marked by expression of a typical estrus sign, that is, standing to be mounted behavior (standing estrus). The onset of estrus was further confirmed by reproductive tract examination (uterine horn tonicity, cervical relaxation, tumefaction of vulva, hyperemia, or reddening of the vulvar mucous membrane), biochemical parameters (cervicovaginal mucus crystallization, fluidity, and spinnbarkeit value (Verma et al., 2014)), and progesterone hormone estimation in blood serum. Transrectal ultrasonography (USG) using a real-time B-mode scanner equipped with a 7.5 MHz rectal probe was used to confirm further presence of dominant follicle (DF; ≥ 10 mm) and absence of CL during the follicular phase (proestrus and estrus) and the onset of ovulation and presence of CL during the luteal phase. Progesterone hormone concentration was estimated in serum samples using an ELISA kit (Cayman Chemical, United States) to confirm the stages of the estrous cycle.

### 2.3 Sample Collection and Processing

Saliva samples were collected from the lower jaw of the mouth of the animal by the direct aspiration method using a 20-ml syringe without a needle in sterile polypropylene tubes (Thermo Fisher Scientific, NY). All the sampling was performed in the early morning (6–7 AM) before feeding, and samples after collection were immediately transported to the laboratory on ice. Samples were centrifuged at 12,000 rpm at 4°C for 30 min to eliminate any particulate matter or feed debris. Supernatant was collected in another 1.5-ml Eppendorf tube, and protease and phosphatase inhibitor (1 × MS-SAFE; Sigma-Aldrich, United States) was added to prevent protein degradation and stored at −80°C until further analysis. Saliva samples from animals wherein estrus and non-estrus stages confirmed through various methods were selected and processed further for proteomics analysis.

### 2.4 Quantification and SDS-PAGE Profiling of Saliva Proteins During Different Stages of the Estrous Cycle

Out of 15 buffaloes, seven animals showing all the cardinal signs of estrus confirmed by gynecological examination, USG, cervicovaginal mucus (CVM) parameters, and progesterone estimation were selected for further proteomics study. The protein concentration of neat saliva samples from PE (*n* = 7), E (*n* = 7), ME (*n* = 7), and DE (*n* = 7) stages were determined with a Bradford assay. Then, salivary proteins, that is, 25 µg from all stages of the estrous cycle, were taken, and initial protein profiling was carried using SDS-PAGE. Initially, proteins were resolved on a 4% stacking and 12% separating acrylamide–bis-acrylamide gel at 15 mA for 3 h using the mini-protean vertical electrophoresis system (Bio-Rad, United States). The gels were stained with Coomassie brilliant blue G (CBB) at room temperature for 1 h, de-stained for 2 h, and scanned using an Epson scanner (GE, Healthcare, United States). The initial protein profiles were identified by running the saliva proteins with a reference protein marker (RTU-BLUeye prestained protein ladder). Based on the intactness and uniformity of the bands, saliva samples from PE, E, ME, and DE stages of five animals were selected for further in-solution digestion and label-free quantification (LFQ) and labeled quantitation using TMT labels and LC-MS/MS analysis.

### 2.5 Label-Free Quantitation

#### 2.5.1 Trypsin In-Solution Digestion of Proteins for LC-MS/MS Analysis

Neat saliva protein of 50 µg from PE, E, ME, and DE stages were taken from five animals, and all samples were processed separately for in-sol digestion. In brief, samples were dissolved in dissolution buffer (50 mM ammonium bicarbonate) and reduced with 50 mM dithiothreitol (Sigma-Aldrich, United States) at 50°C for 45 min. Further alkylation was carried out using 100 mM IAA at room temperature in the dark for 15 min. Trypsin digestion (Promega, Madison, WI) at 1:20 (enzyme: protein) of protein samples was carried out at 37°C for overnight, followed by quenching with 10% trifluoroacetic acid. Furthermore, tryptic-digested samples were vacuum-dried (SpeedVac concentrator, Thermo Fisher Scientific, United States) and desalted using Pierce^®^ C18 Spin Columns (Thermo Fisher Scientific, United States). Desalted peptide samples were vacuum-dried using SpeedVac and reconstituted in 0.1% formic acid. Finally, all 20 samples from five animals of four stages were subjected to LC-MS/MS analysis.

### 2.6 Labeled Quantitation Using TMT Labels

Saliva proteins (75 µg) from each stages of the estrous cycle, that is, PE, E, ME, and DE from five animals were pooled. Proteins were dissolved in dissolution buffer (50 mM ammonium bicarbonate), followed by reduction (50 mM dithiothreitol) at 50°C for 45 min s. Alkylation of cysteine residues was carried out using 100 mM IAA at room temperature in the dark for 15 min s, followed by trypsin digestion at 1:20 (enzyme: protein) at 37°C for overnight. Peptides of PE, E, ME, and DE stages were labeled with 126, 127, 128, and 129 TMT labels, respectively, using TMT 6-plex (Thermo Fisher Scientific, Germany), according to the manufacturer’s protocol. Peptides labeled with TMT tags were incubated for 3 h, quenched (5% hydroxylamine), vacuum-dried, and fractionated into eight fractions using a Pierce™ high pH reversed-phase peptide fractionation kit (Thermo Fisher Scientific, China), according to the manufacturer’s protocol. Furthermore, the fractions were vacuum-dried and reconstituted in 0.1% formic acid and subjected to LC-MS/MS analysis.

### 2.7 ESI–LC-MS/MS Analysis

Peptide samples were analyzed using an EASY-nLC 1,000 mass spectrometer (Thermo Fisher Scientific, United States) coupled with Thermo Fisher Q Exactive equipped with nano-ESI. The peptide mixture of 1 µg was resolved using a 25-cm PicoFrit column (360 µm outer diameter, 75 µm inner diameter, and 10 µm tip) filled with 1.8 µm of C18 resin (Dr. Maisch, Germany). The peptides were loaded with buffer A and eluted with a 0–40% gradient of buffer B (95% ACN and 0.1% formic acid) at a 300 nl/min flow rate for 100 min. MS data were acquired using a data-dependent top 10 method dynamically choosing the most abundant precursor ions from the survey scan.

### 2.8 Data Processing

The MS/MS data were searched and analyzed with Proteome Discoverer (v2.2) against the UniProt *Bos taurus* reference proteome database. In Proteome Discoverer, the Sequest search engine was used; the precursor and fragment mass tolerances were set at 10 ppm and 0.5 Da, respectively. The protease used to generate peptides, that is, enzyme specificity, was set at trypsin/P (cleavage at the C terminus of “K/Rˮ: unless followed by “Pˮ) along with a maximum missed cleavages value of two. The “ion score cutoff” was set to 20, thereby eliminating the lowest quality matches and minimum peptide length as six amino acid residues. Carbamidomethyl on cysteine was set at fixed modification and oxidation of methionine, and N-terminal acetylation was considered variable modifications for database search. The maximal number of modifications per peptide was set at 6. The minimum peptide length parameter was set to 6, and the “peptide” re-quantification function was enabled. Both peptide spectra match, and the protein false discovery rate (FDR) was set to 0.01. The decoy-reversed sequences database was included for the calculation of the FDR.

### 2.9 Bioinformative Analysis and Network Generation

Protein class analysis of the differentially expressed proteins based on molecular function, biological process, and cellular component was performed using Cytoscape ClueGo plug-in (v.3.7.2). A protein–protein interaction (PPI) network was constructed with the Search Tool for the Retrieval of Interacting Genes (STRING) database (v.11.5) and Cytoscape. A hypergeometric test (*p* < 0.05) was used in statistical analysis in the ClueGO tool. Furthermore, the online KEGG (Kyoto Encyclopedia of Genes and Genomes) pathway database was used for biological interpretation of higher-level systemic functions.

### 2.10 Western Blot Analysis

A quantity of 40 µg salivary proteins from four stages was loaded in a 12% SDS-PAGE gel and then transferred onto a polyvinylidene difluoride (PVDF) membrane using a semidry Western blot transfer system (Invitrogen, United States). The membrane was incubated with 5% BSA in TBST buffer (10 mM Tris, 150 mM NaCl, 0.05% Tween 20, and 5% BSA, pH 7.60) as blocking solution overnight at 4°C. After blocking, the membrane was incubated separately with seven primary antibodies: SERPINB1 (Aviva Systems Biology, San Diego, United States; 1:5,000), HSPA1A (Sigma-Aldrich, United States; 1:5,000), ENO3 (Sigma-Aldrich, MO, United States; 1:1,250), LCN1 (MyBiosource, CA, United States; 1:1,000), SDF4 (Aviva Systems Biology, San Diego, United States; 1:5,000), VMO1 (Thermo Fischer Scientific, United States; 1:5,000), and beta-actin (Sigma-Aldrich, MO, United States; 1:1,667) at room temperature for 1 h. Subsequently, the membranes were washed three times with TBST followed by incubation with horseradish peroxidase–conjugated secondary antibody (diluted 1:4,000, Sigma-Aldrich, United States) for 1 h at room temperature. The membranes were dried and developed using an X-ray film with Clarity Western ECL substrate (Bio-Rad, United States). The band intensity of the protein expression was normalized with beta-actin as an internal control, and values were analyzed using one way ANOVA with SPSS Inc (2007) after normalization.

## 3 Results

### 3.1 Estrus Detection and Confirmation

**FIGURE 1:**
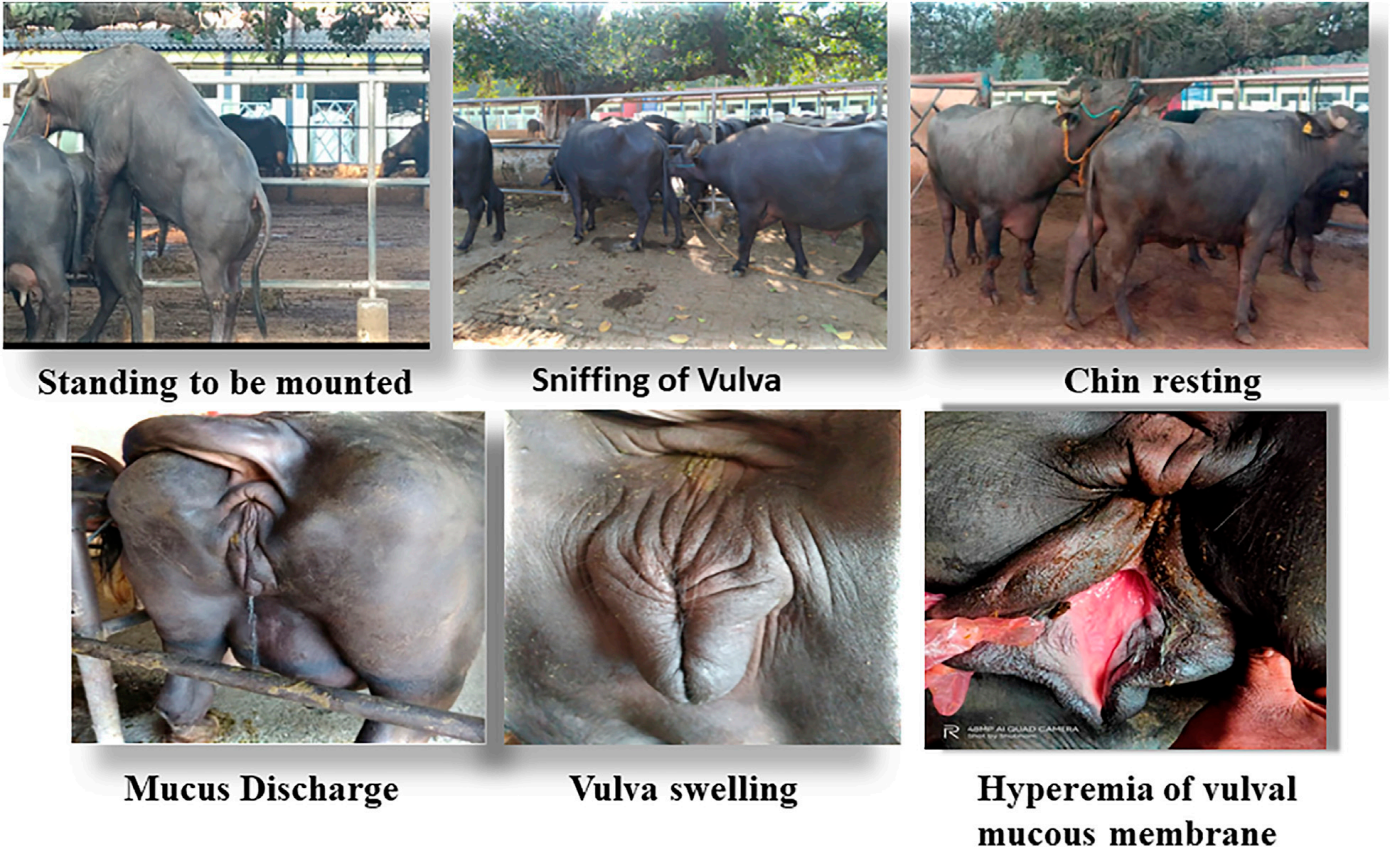
Behavioral signs of estrus in buffaloes.

**FIGURE 2:**
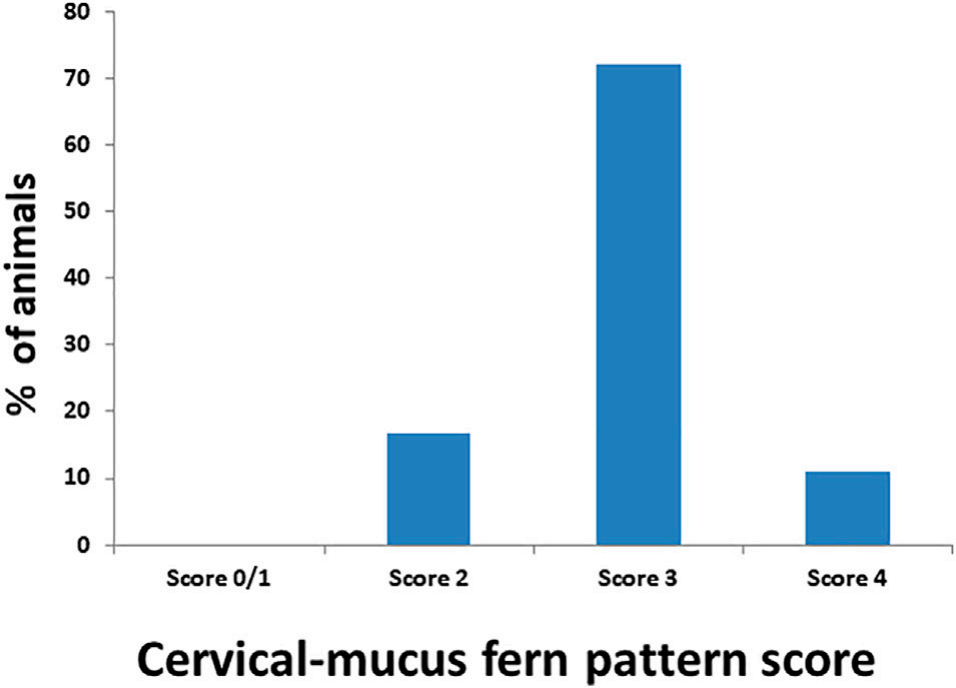
Estimation of the crystallization/fern pattern score of cervicovaginal mucus during estrus in buffaloes.

**FIGURE 3:**
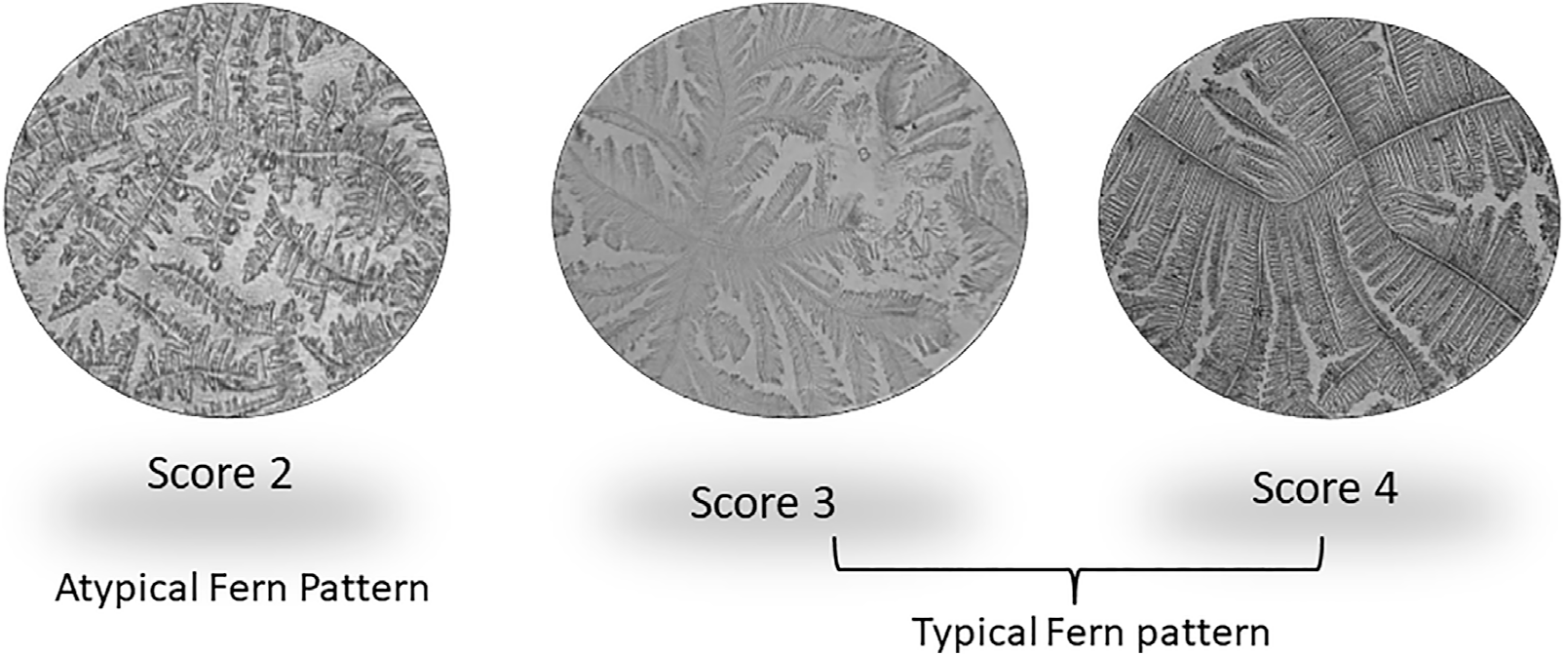
Different crystallization/fern patterns of cervicovaginal mucus observed during estrus in buffaloes.

**FIGURE 4:**
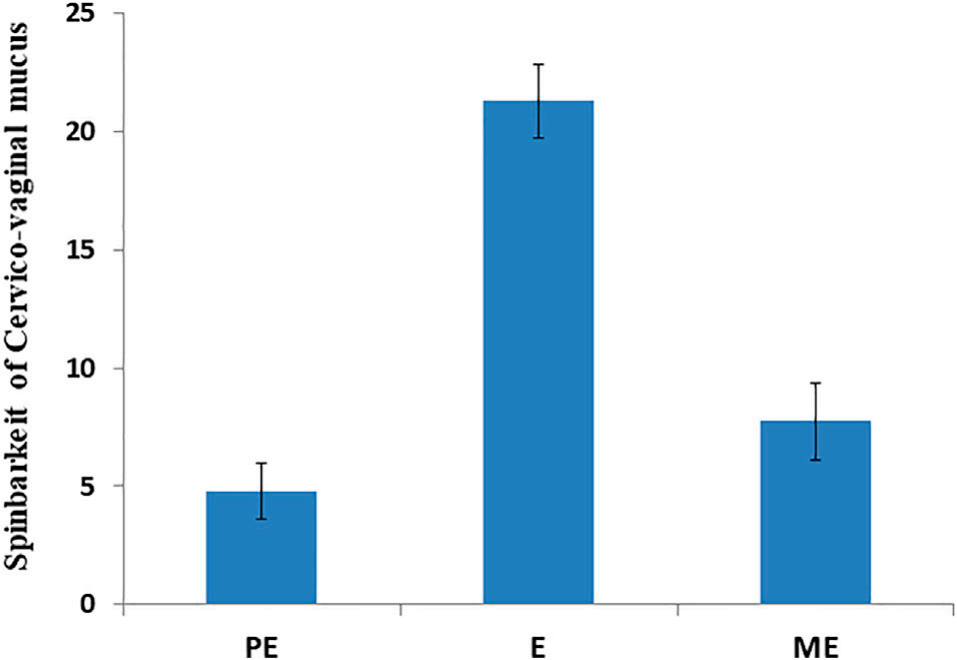
Spinnbarkeit value of cervicovaginal mucus during proestrus (PE), estrus (E), and metestrus (ME) stages of the buffalo estrous cycle.

**FIGURE 5:**
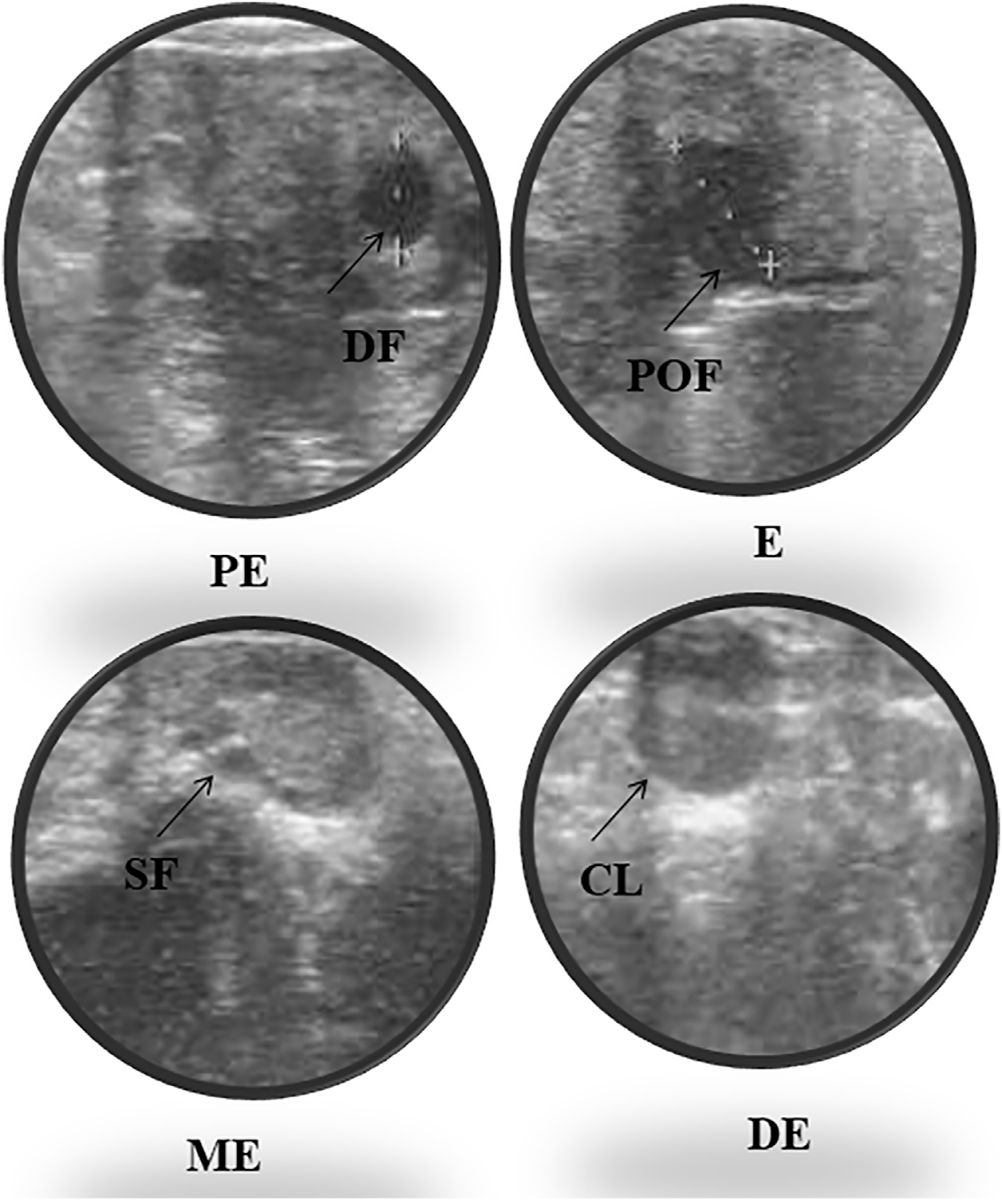
Ultrasonography of ovarian follicles and corpus luteum (CL) during proestrus (PE), estrus (E), metestrus (ME), and diestrus (DE) stages of the buffalo estrous cycle (DF, dominant follicle; SF, small follicle; POF, preovulatory follicle; CL, corpus luteum).

**FIGURE 6:**
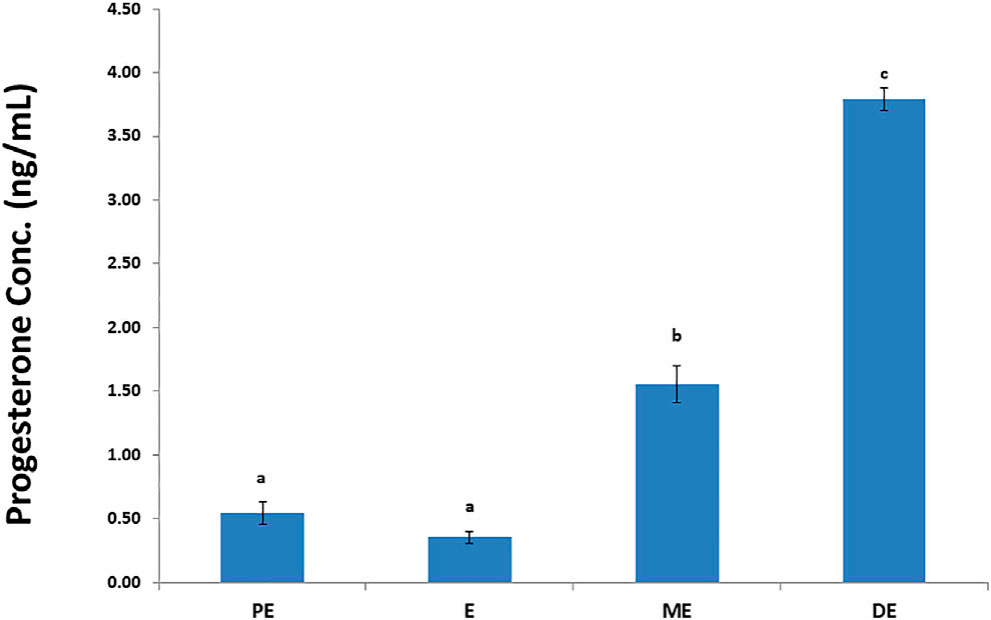
Progesterone hormone concentration (ng/ml) during proestrus (PE), estrus (E), metestrus (ME), and diestrus (DE) stages of the buffalo estrous cycle.

Accurate and efficient identification of estrus is essential for successful conception and efficient breeding management of farm animals including cattle and buffaloes. Hence, the onset of estrus in buffaloes was determined by physical signs and confirmed by gynecological examination of reproductive tract, transrectal USG, biochemical parameters (CVM fern pattern, spinnbarkeit value, and other parameters), and progesterone hormone estimation in blood serum. Behavioral signs of estrus are listed in Table 1; Figure 1. Major estrus signs exhibited by female buffaloes were cervicovaginal mucus discharge (89.9%), standing to be mounted (83.3%), bellowing (77.7%), frequent micturition (65.1%), hyperemia of the vulval mucous membrane (89.9%), and vulva tumefaction (72.2%). Further male behavior toward estrus buffaloes such as flehmen’s reaction (81.1%), sniffing the vulva (71.1%), and chin resting (68.2%) were also considered for estrus identification. Other estrus signs such as uterine horn tonicity, CVM crystallization, and spinnbarkeit value were considered for confirmation of estrus. Tonicity of uterine horns (94%) was mostly intense during the estrus stage (Table 2). The CVM crystallization/fern pattern was typical with scores 3 and 4 in 83.3% during estrus (Figures 2, 3). The spinnbarkeit value of CVM was the highest (21.30 ± 1.56 cm) during estrus (Figure 4). Transrectal USG revealed the presence of dominant follicle (DF) during the follicular phase, and the size of DF was 12.1 ± 0.03 mm and 13.5 ± 0.04 mm during PE and E, respectively (Figure 5). The absence of preovulatory follicle confirmed ovulation during the ME stage and presence of CL during the DE stage (Figure 5). The serum progesterone level was found to be lowest during E (0.35 ± 0.04 ng/ml, *p* < 0.05) and PE (0.54 ± 0.08 ng/ml) than that in ME (1.56 ± 0.14 ng/ml) and DE (3.79 ± 0.09 ng/ml) stages (Figure 6).

**TABLE 1:** Behavioral signs of estrus in buffaloes.

Behavioral signs of estrus	% of animals showing estrus signs
Standing to be mounted	83.3
Cervicovaginal mucus discharge	89.9
Hyperemia of the vulval mucous membrane	89.9
Flehmen’s response	81.1
Bellowing	77.7
Vulva tumefaction	72.2
Frequent micturition	65.1
Licking/sniffing of vulva	71.1
Chin resting	68.2

**TABLE 2:** Intensity of expression of other estrus signs in buffaloes.

Estrus sign	Intensity of expression (%)		
	Mild	Moderate	Intense
Uterine horn tonicity	-	6	94
Vulva tumefaction	-	27.8	72.2
Hyperaemia/reddening of the vulval mucous membrane	-	10.1	89.9
Cervicovaginal mucus discharge	-	16.7	83.3

### 3.2 Quantification and SDS-PAGE of Salivary Proteins

**FIGURE 7:**
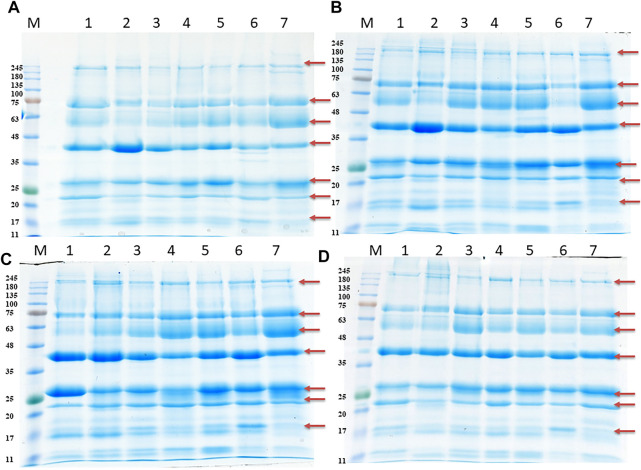
SDS-PAGE of salivary proteins during proestrus (PE) **(A)**, estrus (E) **(B),** metestrus (ME) **(C),** and diestrus (DE) **(D)** stages of the buffalo estrous cycle. M, protein molecular markers; Lane 1-7 contains 25 μg protein from 7 individual buffaloes. Arrow indicates presence of 7 major bands.

The salivary proteins were quantified, and the concentration was in the range of 500–1,000, 400–1,000, 400–900, and 466–800 μg/ml during PE, E, ME, and DE stage, respectively. Furthermore, salivary proteins were separated using SDS-PAGE. A total of seven major and several minor bands were exhibited in the CBB-stained gel invariably among four stages of the estrous cycle and their molecular mass ranged from 17 to 245 kDa (Figure 7).

### 3.3 LC-MS/MS Analysis and DEPs Comparisons

The LFQ-LC-MS/MS analysis identified 520 DEPs with 1% protein and peptide FDR cutoff. The detailed information related to accession, description, protein FDR confidence, peptide score, coverage %, peptides, PSM, unique peptides, protein groups, MW, calculated pI, Sequest score, gene name, and abundance of the identified proteins is shown in Supplementary Table S1. The number of upregulated and downregulated proteins was distinctly identified based on the fold-change criteria between E, PE, ME, and DE stages. The fold-change values were set between 0.5 and 1.5; the proteins expressed with values greater than 1.5 were upregulated, and the proteins expressed with values less than 0.5 were downregulated. The list of upregulated and downregulated proteins during E compared to other non-estrus stages (PE, ME, and DE) is listed in Table 3. By considering the fold-change range, a total of 59 proteins and four proteins were found to be upregulated and downregulated during E compared to PE, ME, and DE stages, respectively. Among the DEPs, few proteins with higher abundance at E than all other non-estrus stages and identified in all the biological replicate samples were gastric triacylglycerol lipase (LIPF; E/P: 33-fold, E/M: 38.8-fold, and E/D: 19-fold), peptidyl-prolyl cis-trans isomerase E (PPIE; E/P: 21-fold, E/M: 15-fold, and E/D: 25-fold), fatty acid–binding protein, (FABP3; E/P: 2.4-fold, E/M: 6.2-fold, and E/D: 10.8-fold), leukocyte elastase inhibitor (SERPINB1; E/P: 3-fold, E/M: 2.3-fold, and E/D: 3.4-fold), apolipoprotein A-I (APOA1; E/P: 1.9-fold, E/M: 9.7-fold, and E/D: 6.8-fold), heat shock 70-kDa protein 1A (HSPA1A; E/P: 1.5-fold, E/M: 2.3-fold, and E/D: 3.6-fold), and pyruvate kinase (PKLR; E/P: 5-fold, E/M: 2-fold, and E/D: 4.7-fold) were highly abundant proteins during E vs. PE, ME, and DE comparisons.

**TABLE 3:** List of differentially expressed proteins (DEPs) during estrus (E) vs. proestrus (PE), metestrus (ME), and diestrus (DE) stages using LFQ-LC-MS/MS analysis.

Accession	Gene symbol	Description	Peptide	Fold change	Fold change	Fold change
				E/PE	E/ME	E/DE
F1MES3	LIPF	Gastric triacylglycerol lipase	4	33.125	38.834	18.989
Q29458	LIPF	Gastric triacylglycerol lipase	7	32.65	39.416	19.173
A4FV72	PPIE	Peptidyl-prolyl cis–trans isomerase E	1	21.071	15.047	24.834
P00639	DNASE1	Deoxyribonuclease-1	2	7.005	4.783	10.895
F1N0N9	—	Uncharacterized protein	1	5.447	1.582	11.299
P0CG53	UBB	Polyubiquitin-B	1	2.301	10.493	3.44
P10790	FABP3	Fatty acid-binding protein, heart	1	2.445	6.187	10.788
P02690	PMP2	Myelin P2 protein	1	2.445	6.187	10.788
P15497	APOA1	Apolipoprotein A-I	6	1.942	9.793	6.824
G3MZU3	LOC783399	Uncharacterized protein	4	1.175	8.238	5.386
Q865V6	CAPG	Macrophage-capping protein	1	5.134	1.633	4.546
Q1JPG7	PKLR	Pyruvate kinase	1	5.05	2.016	4.72
G3N2P2	LOC613345	Uncharacterized protein	2	4.503	2.859	2.129
P19661	CATHL3	Cathelicidin-3	2	4.339	2.036	2.882
Q2KJF1	A1BG	Alpha-1B-glycoprotein	1	4.135	1.934	3.79
P02070	LOC100850059; HBB	Hemoglobin subunit beta	13	3.95	1.725	1.372
P02313	HMGN2	Non-histone chromosomal protein HMG-17	1	3.74	3.175	2.366
P19660	CATHL2	Cathelicidin-2	3	3.573	3.92	4.087
Q2KIT0	MGC137014	Protein HP-20 homolog	1	3.457	3.269	2.615
G1K1L8	SERPINB1	Leukocyte elastase inhibitor	7	3.116	2.307	3.904
Q1JPB0	SERPINB1	Leukocyte elastase inhibitor	10	3.01	2.349	3.419
P79125	BPIFA2B	Short palate, lung and nasal epithelium carcinoma–associated protein 2B	1	2.938	2.139	1.437
P54229	CATHL5	Cathelicidin-5	3	2.561	1.439	1.781
P56425	CAMP	Cathelicidin-7	2	2.351	2.847	1.202
Q0VCX4	CTNNB1	Catenin beta-1	1	2.348	5.891	2.679
Q3ZC09	ENO3	Beta-enolase	3	2.302	0.7	4.444
A5D984	PKM	Pyruvate kinase	11	2.293	1.513	1.65
Q0VCW4	SDS	l-Serine dehydratase/l-threonine deaminase	6	2.233	1.876	1.996
F1N0R8	SDS	l-Serine dehydratase/l-threonine deaminase	6	2.233	1.876	1.996
P00975	—	Serum basic protease inhibitor	1	2.154	1.683	1.943
Q148F1	CFL2	Cofilin-2	1	2.097	1.326	1.337
F1MLW2	BPIFB1	BPI fold-containing family B member 1	10	2.011	1.322	2.338
F1MKB7	GSTO1	Uncharacterized protein	2	1.859	1.86	4.825
A5PJE3	FGA	Fibrinogen alpha chain	1	1.821	5.715	5.472
P02672	FGA	Fibrinogen alpha chain	1	1.821	5.715	5.472
P54228	CATHL6	Cathelicidin-6	4	1.785	2.494	1.382
A6QR19	ENO2	ENO2 protein	2	1.779	1.309	1.637
P02754	PAEP	Beta-lactoglobulin	10	1.703	1.446	1.519
G5E5H7	PAEP	Uncharacterized protein	9	1.703	1.732	1.371
E1BI89	—	Uncharacterized protein	13	1.682	1.539	3.423
P02676	FGB	Fibrinogen beta chain	1	1.568	7.645	6.275
F1MBN5	FOLR1; FOLR3	Folate receptor alpha	3	1.526	2.006	0.975
P0CB32	HSPA1L	Heat shock 70-kDa protein 1-like	1	1.474	2.283	3.613
Q27975	HSPA1A	Heat shock 70-kDa protein 1A	1	1.474	2.283	3.613
Q27965	HSPA1A	Heat shock 70-kDa protein 1B	1	1.474	2.283	3.613
G3X701	LOC515966	Uncharacterized protein	2	1.44	1.708	2.409
P05307	P4HB	Protein disulfide-isomerase	1	1.393	1.54	1.98
Q0VCN9	FOLR2	Folate receptor 2 (Fetal)	1	1.384	2.006	2.372
G3MX65	WFDC2	Uncharacterized protein	2	1.322	2.324	3.6
G3X8G9	—	Uncharacterized protein	6	1.311	2.365	1.463
Q865S1	AP3D1	AP-3 complex subunit delta-1	1	1.285	7.378	5.363
Q2TBU0	HP	Haptoglobin	14	1.283	1.481	2.495
A6H6Z5	GALNT6	Polypeptide N-acetylgalactosaminyl transferase 6	3	1.271	1.388	1.416
E1BBX7	LCNL1	Uncharacterized protein	2	1.24	2.655	8.647
G5E5A7	—	Uncharacterized protein	34	1.142	1.536	1.602
G3MX66	VMO1	Uncharacterized protein	6	1.074	1.743	2.984
F1MK50	OBP2B	Uncharacterized protein	3	1.056	1.341	2.985
G3X799	OBP	Uncharacterized protein	9	0.944	1.437	1.674
Q8SPU5	BPIFA1	BPI fold-containing family A member 1	4	0.791	5.438	5.007
**Downregulated protein**						
G3MZ19	LOC100295741; ZG16B	HRPE773-like	8	0.445	0.463	0.134
A6QLQ8	ENDOU	Poly(U)-specific endoribonuclease	2	0.453	0.364	0.433
A4IFU5	HIST3H2A	Histone H2A	3	0.43	0.443	0.539
G5E5V1		Uncharacterized protein	2	0.56	0.306	0.581

Similarly, TMT-LC-MS/MS analysis identified a total of 369 DEPs; among these 74 and 73 proteins were upregulated and downregulated during E compared to PE, ME, and DE stages, respectively. The detail information of identified proteins is listed in Supplementary Table S2. The list of upregulated and downregulated proteins during E than other non-estrus stages (PE, ME, and DE) is presented in Tables 4, 5, respectively. Among the upregulated DEPs, odorant-binding protein (OBP; E/P: 2.5-fold, E/M: 2.5-fold, and E/D: 69-fold), lipocalin 1 (LCN1; E/P: 7.3-fold, E/M: 14.3-fold, and E/D: 11.3-fold), odorant-binding protein-like (MGC151921; E/P: 1.8-fold, E/M: 2.8-fold, and E/D: 24-fold), lipocln_cytosolic_FA-bd_dom domain-containing protein (LOC104969973; E/P: 11.3-fold, E/M: 14.6-fold, and E/D: 5.8-fold), heat shock protein beta-1 (HSPB1; E/P: 12.5-fold, E/M: 15.5-fold, and E/D: 7.3-fold), galectin (LGALS7B; E/P: 5.3-fold, E/M: 6.6-fold, and E/D: 4.6-fold), metalloproteinase inhibitor 2 (TIMP2; E/P: 11.2-fold, E/M: 11.9-fold, and E/D: 2.7-fold), 45-kDa calcium-binding protein (SDF4; E/P: 62.6-fold, E/M: 88.6-fold, and E/D: 19.2-fold), peptidyl-prolyl isomerase (LOC526524; E/P: 13.6-fold, E/M: 17.3-fold, and E/D: 37.9-fold), and vitelline membrane outer layer 1 (VMO1; E/P: 62.6-fold, E/M: 88.6-fold, and E/D: 19.2-fold) were highly abundant proteins during E than other non-estrus stages, and few of these were also discovered in our previous investigation.

**TABLE 4:** List of upregulated proteins during estrus (E) vs. proestrus (PE), metestrus (ME), and diestrus (DE) stages using TMT-LC-MS/MS analysis.

Accession	Gene symbol	Description	Peptide	Fold change E/PE	Fold change E/ME	Fold change E/DE
F1MKI5	SDF4	45-kDa calcium-binding protein	2	71.71	100	19.73
Q3ZBZ1	SDF4	45-kDa calcium-binding protein	3	62.663	88.648	19.262
Q2NKS8	LOC526524	Peptidyl-prolyl isomerase	1	13.678	17.355	37.984
E1BKA1	LOC786350	Protein S100	1	14.447	19.419	1.34
G3MZ21	—	Uncharacterized protein	1	13.057	8.159	2.728
E1BEL8	HBE1	Globin B1	1	13.057	8.159	2.728
E1BEL7	HSPB1	Heat shock protein beta-1	6	12.524	15.547	7.289
Q58DP7	HSPB1	Heat shock 27-kDa protein 1	5	12.524	15.547	7.289
G3X7S2	HSPB1	Heat shock protein beta-1	5	12.524	15.547	7.289
Q5KR47	TPM3	Tropomyosin alpha-3 chain	1	11.813	13.017	1.782
A6QR15	LOC535277	LOC535277 protein	1	11.813	13.017	1.782
Q5KR48	TPM2	Tropomyosin beta chain	1	11.813	13.017	1.782
Q5KR47-2	TPM3	Isoform 2 of Tropomyosin alpha-3 chain	1	11.813	13.017	1.782
P81947	TUBA1B; TUBA1A	Tubulin alpha-1B chain	2	11.444	12.223	5.148
F1MNF8	LOC100141266	Tubulin alpha chain	1	11.444	12.223	5.148
G3X700	LOC104969973	Uncharacterized protein	7	11.348	14.639	5.809
Q862B8	TIMP2	Similar to metalloproteinase inhibitor	3	11.285	11.992	2.762
P07435	OBP	Odorant-binding protein	8	2.573	2.481	69.029
Q862Q0	PGAM1	Phosphoglycerate mutase	1	3.695	5.268	48.354
Q0IIA2	MGC151921	Odorant-binding protein-like	5	1.784	2.753	23.736
A5PJH7	LOC788112	LOC788112 protein	1	2.292	1.987	21.114
A8KC76	HSPA8	HSPA8 protein	1	4.673	4.937	18.413
Q865V6	CAPG	Macrophage-capping protein	1	3	3.531	14.154
Q3ZCL8	SH3BGRL3	SH3 domain-binding glutamic acid-rich-like protein 3	2	2.104	2.295	12.421
F1MS23	LCN1	Lipocalin 1	6	7.354	14.39	11.301
P02070	HBB	Hemoglobin subunit beta	9	9.977	6.41	2.243
F1MKC4	—	Uncharacterized protein	5	9.876	9.214	2.063
Q58DT9	ACTA2	Alpha 2 actin	9	9.561	8.913	2.069
G8JKX4	ACTA2	Actin, aortic smooth muscle	9	9.561	8.913	2.069
E1BLR9	CPD	Carboxypeptidase D	1	8.519	11.821	3.381
P60712	ACTB	Actin, cytoplasmic 1	17	8.154	7.925	2.061
F1MRD0	ACTB	Actin, cytoplasmic 1	14	8.154	7.925	2.061
P81265-2	PIGR	Isoform short of polymeric immunoglobulin receptor	15	7.975	8.284	5.803
G5E6M1	PIGR	Polymeric immunoglobulin receptor	14	7.975	8.284	5.803
G3N2H5	S100A12	Protein S100	1	7.311	3.613	2.133
A0A1C9EIX6	HSPB1	Heat shock protein family B member 1 variant 2	7	6.972	7.193	5.125
F1N650	ANXA1	Annexin	2	6.562	4.819	0.855
Q712W6	GAPDH	Glyceraldehyde 3-phosphate dehydrogenase	3	5.445	4.678	0.97
E1BDE6	LGALS7B	Galectin	6	5.339	6.629	4.633
G3N3D0	LGALS7	Galectin	6	5.339	6.629	4.633
G3MX66	VMO1	Vitelline membrane outer layer 1 homolog	5	5.3	5.5	4.1
P81265	PIGR	Polymeric immunoglobulin receptor	29	5.127	5.514	4.049
A6QNW3	PIGR	PIGR protein	29	5.127	5.514	4.049
G5E5C8	TALDO1	Transaldolase	3	4.986	4.795	1.281
A5D7E8	PDIA3	Protein disulfide-isomerase	2	4.969	3.617	1.212
E1B970	GOLGA3	Golgin A3	1	4.283	5.078	6.458
A6QQ07	BTD	Biotinidase	1	3.945	3.735	2.313
Q0P569	NUCB1	Nucleobindin-1	9	3.793	3.702	4.757
P68252	YWHAG	14-3-3 protein gamma	2	3.757	4.645	9.328
G3X894	LOC786263	Uncharacterized protein	5	3.71	5.005	0.897
Q629I2	RPVgp5	Fusion glycoprotein F0	1	3.705	4.751	3.904
P05307	P4HB	Protein disulfide-isomerase	3	3.338	3.649	9.38
Q3SZ62	PGAM1	Phosphoglycerate mutase 1	6	3.21	3.424	3.828
G3X799	OBP	Uncharacterized protein	9	3.041	2.813	2.032
F1N614	HSPA5	78-kDa glucose-regulated protein	2	3.018	3.507	2.316
Q2KJF1	A1BG	Alpha-1B-glycoprotein	2	2.625	2.919	2.931
Q3T145	MDH1	Malate dehydrogenase, cytoplasmic	2	2.547	2.689	1.172
F1N3A1	THBS1	Thrombospondin-1	10	2.524	2.7	0.95
F1MKE7	KRT6C	Uncharacterized protein	9	2.385	3.018	4.091
Q3T010	PEBP4	Phosphatidylethanolamine-binding protein 4	2	2.212	3.24	2.012
F2FB41	MUC5AC	Mucin-5AC	6	2.197	3.168	1.523
Q3SZH5	AGT	Angiotensinogen	4	1.98	1.909	2.112
F2Z4I6	HIST2H2AC	Histone H2A	5	1.928	2.208	2.153
A1A4R1	HIST2H2AC	Histone H2A type 2-C	5	1.928	2.208	2.153
F1MRN2	—	Histone H2A	3	1.928	2.208	2.153
F1MLQ1	LOC524236	Histone H2A	2	1.928	2.208	2.153
Q862L0	ACTG2	Similar to beta-actin	4	1.745	1.596	3.548
F1MB90	OVOS2	Uncharacterized protein	16	1.726	2.819	1.215
F6QEL0	—	Cystatin	2	1.427	1.338	3.907
Q5DPW9	CST6	Cystatin	2	1.427	1.338	3.907
F1MVR7	FAM25A	Uncharacterized protein	1	1.378	1.946	1.935
E1BI89	LOC101908058	Uncharacterized protein	4	1.355	1.541	2.142
K4JDT2	A2M	Alpha-2-macroglobulin variant 20	12	1.243	2.308	8.216
F1N4C3	BPIFA2B	Uncharacterized protein	3	0.428	0.285	1.713

**TABLE 5:** List of downregulated proteins during estrus (E) vs. proestrus (PE), metestrus (ME), and diestrus (DE) stages using TMT-LC-MS/MS analysis.

Accession	Gene symbol	Description	Peptide	Fold change E/PE	Fold change E/ME	Fold change E/DE
B9TUD2	CATHL7	Cathelicidin-7	1	0.01	0.01	0.01
G3N1R1	—	Uncharacterized protein	1	0.01	0.01	0.063
B9TUC3	CATHL2	Cathelicidin-2	1	0.01	0.01	0.01
Q148E6	CYP4B1	Cytochrome P450 family 4 subfamily B member 1	1	0.01	0.01	0.234
Q1RMN8	IGL@	Immunoglobulin light chain, lambda gene cluster	14	0.014	0.012	0.228
Q28133	BDA20	Allergen Bos d 2	2	0.019	0.095	0.045
A5D7Q2	LOC524810	Uncharacterized protein	9	0.021	0.023	0.112
K4JR84	A2M	Alpha-2-macroglobulin variant 17	2	0.021	0.038	0.393
E1BI82	LOC525947	Uncharacterized protein	1	0.022	0.028	0.275
G3X6K8	HP	Haptoglobin	3	0.028	0.057	0.069
Q3ZCL0	CRISP3	Cysteine-rich secretory protein 2	3	0.032	0.038	0.021
F6R3I5	CRISP3	Uncharacterized protein	4	0.032	0.038	0.021
P02702	FOLR1; FOLR3	Folate receptor alpha	1	0.039	0.049	0.586
E1B8Q6	LOC524080	Uncharacterized protein	1	0.044	0.045	0.01
F1MD73	—	Uncharacterized protein	7	0.045	0.054	0.133
P60986	PIP; LOC107131147	Prolactin-inducible protein homolog	10	0.047	0.062	0.056
F1MCV8	—	Uncharacterized protein	3	0.047	0.062	0.056
F1N6D1	LOC100847724	Uncharacterized protein	13	0.047	0.069	0.016
G3N269	FABP5	Fatty acid-binding protein, epidermal	3	0.053	0.071	0.136
F1MHQ2	BPIFA2A	Uncharacterized protein	21	0.056	0.047	0.048
Q6PVY3	MIF	Macrophage migration inhibitory factor	1	0.057	0.066	0.067
Q6H320	KLK1	Glandular kallikrein	6	0.058	0.071	0.148
A0JNP2	SCGB1D	Secretoglobin family 1D member	1	0.07	0.117	0.352
G3N2D7	LOC100297192	Uncharacterized protein	4	0.073	0.067	0.128
K4JR71	A2M	Alpha-2-macroglobulin variant 2	4	0.076	0.132	0.206
P17697	CLU	Clusterin	5	0.08	0.08	0.444
E1BL84	—	Uncharacterized protein	2	0.08	0.082	0.013
F1MSB7	PLS3	Plastin-3	9	0.082	0.091	0.036
Q862H7	S100A11	Protein S100	1	0.086	0.075	0.01
O62672	MUC19	Submaxillary mucin 1	9	0.087	0.084	0.428
F1MWN7	—	Uncharacterized protein	9	0.087	0.084	0.431
F1N0W1	FCHSD1	Uncharacterized protein	6	0.091	0.161	0.07
Q5E9B1	LDHB	l-Lactate dehydrogenase B chain	2	0.101	0.083	0.032
G1K1L8	—	Uncharacterized protein	9	0.106	0.117	0.104
A6QQF6	SBSN	Suprabasin	1	0.109	0.112	0.06
A2VE41	EFEMP1	EGF-containing fibulin-like extracellular matrix protein 1	14	0.12	0.099	0.161
E1BAU5	LOC512548; SLPI	Uncharacterized protein	1	0.124	0.171	0.479
A4IFI0	IGLL1	IGLL1 protein	9	0.132	0.115	0.289
F1MCF8	LOC100297192	Uncharacterized protein	11	0.132	0.115	0.289
A5PK49	IGL@	IGL@ protein	6	0.132	0.115	0.289
A6H7J7	LOC100297192	Uncharacterized protein	9	0.139	0.121	0.297
P02253	HIST1H1C	Histone H1.2	18	0.143	0.107	0.05
F2FB39	MUC19	Mucin-19	14	0.154	0.136	0.067
G3X6I0	—	Uncharacterized protein	10	0.172	0.213	0.246
P19858	LDHA	l-Lactate dehydrogenase A chain	21	0.172	0.134	0.032
G3MX67	—	Uncharacterized protein	2	0.176	0.157	0.213
F1MBA5	—	Uncharacterized protein	6	0.176	0.157	0.213
G3N0H7	—	Uncharacterized protein	4	0.176	0.157	0.213
A0A1Y0KDJ6	—	Beta-casein	9	0.177	0.181	0.495
F1MGF6	—	Uncharacterized protein	13	0.181	0.165	0.052
G9G9X6	LALBA	Alpha-lactalbumin protein variant D	14	0.188	0.254	0.352
Q3SYR8	IGJ; JCHAIN	Immunoglobulin J chain	10	0.19	0.166	0.191
B2BX69	AZGP1	Zn-alpha-2-glycoprotein	3	0.197	0.19	0.156
B2BX70	AZGP1	Zn-alpha-2-glycoprotein	2	0.197	0.19	0.156
A5D7V3	ESM1	ESM1 protein	2	0.213	0.297	0.145
G5E513	IGHM	Immunoglobulin heavy constant mu	5	0.219	0.455	0.126
P01035	CST3	Cystatin-C	6	0.221	0.184	0.302
G5E5Q6	TFF3	Trefoil factor 3	3	0.229	0.232	0.22
Q6LC78	LTF	Lactoferrin	8	0.23	0.432	0.369
G5E5T5	—	Uncharacterized protein	8	0.231	0.459	0.424
E1B8Q2	LOC104969118	Uncharacterized protein	5	0.241	0.216	0.775
Q8MII0	LTF	Lactotransferrin	8	0.244	0.388	0.394
F1MB32	A2ML1	Uncharacterized protein	3	0.244	0.318	0.087
Q6LBN7	LTF	Lactoferrin	18	0.254	0.424	0.295
Q3SZ57	AFP	Alpha-fetoprotein	1	0.258	0.427	0.063
Q19KS1	LTF	Lactoferrin	7	0.269	0.484	0.223
Q683R8	Cath	Cathelicidin	3	0.316	0.364	0.079
A6QQA8	QSOX1	Sulfhydryl oxidase	1	0.316	0.392	0.14
P00432	CAT	Catalase	1	0.33	0.385	0.023
P33046	CATHL4; LOC786887	Cathelicidin-4	1	0.377	0.335	0.058
F1MH40	—	Uncharacterized protein	1	0.417	0.43	0.413
X5F5B4	SERPINB4	Serpin B4-like protein	2	0.456	0.557	0.566
A6QPZ4	SERPINB4	SERPINB4 protein	2	0.456	0.557	0.566

**FIGURE 8:**
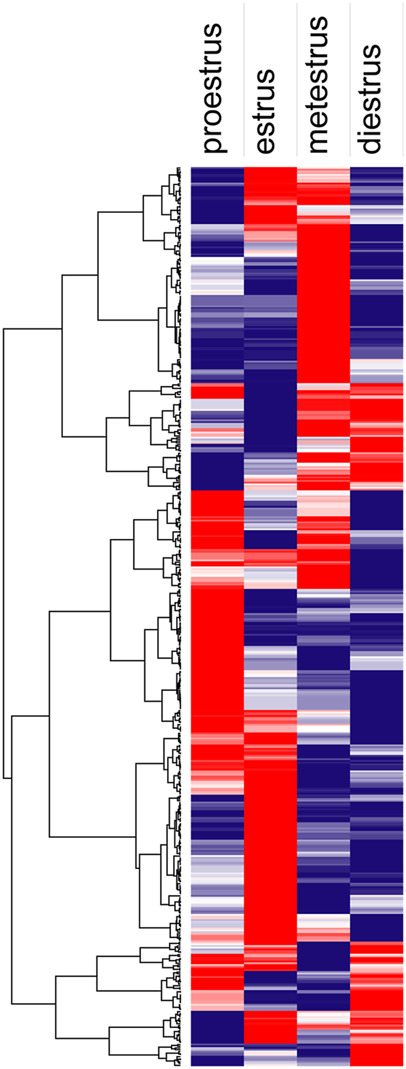
Hierarchical clustering analysis shows proteins identified during proestrus, estrus, metestrus, and diestrus stages (red to blue indicates highest to lowest abundance).

**FIGURE 9:**
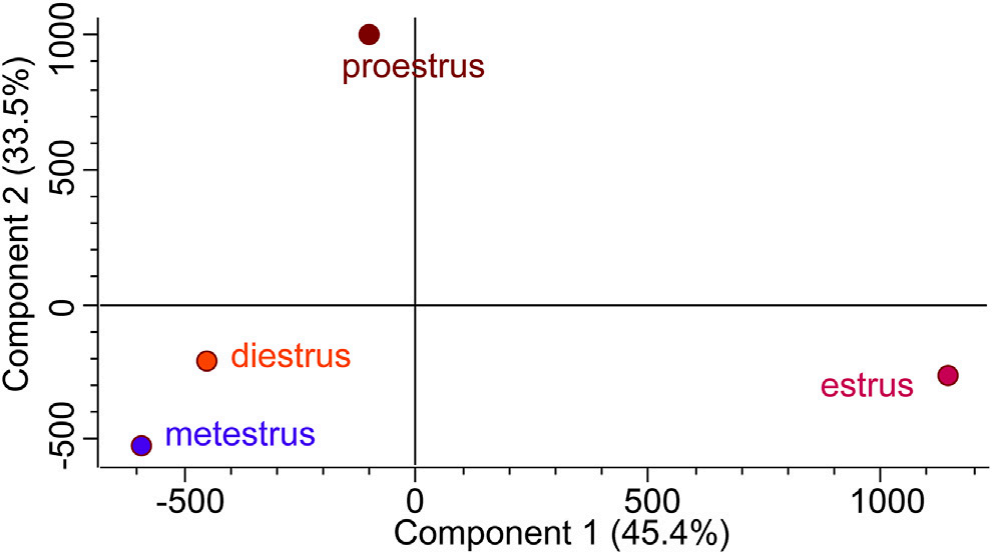
Principal component analysis (PCA) attributes the more difference between estrus and other non-estrus stages (proestrus, metestrus, and diestrus).

**FIGURE 10:**
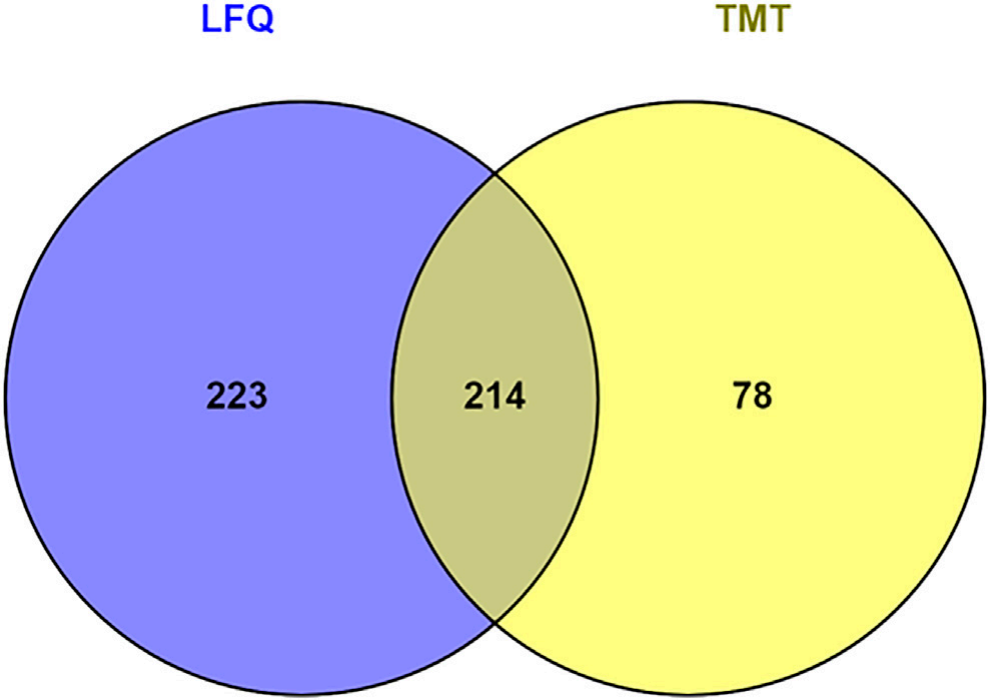
Venn diagram represents number of proteins identified by tandem mass tag (TMT) and label-free quantitation (LFQ).

Hierarchical clustering (Figure 8) and principal component analysis (PCA) (Figure 9) of proteins identified during four stages depicts clear cut differences in the expression of proteins among four stages. A Venn diagram analysis revealed that 214 proteins were commonly identified by both techniques, that is, TMT and LFQ; however, 223 and 78 proteins were specifically identified using LFQ and TMT methods, respectively (Figure 10).

### 3.4 Validation of DEPs Using Western Blotting

**FIGURE 11:**
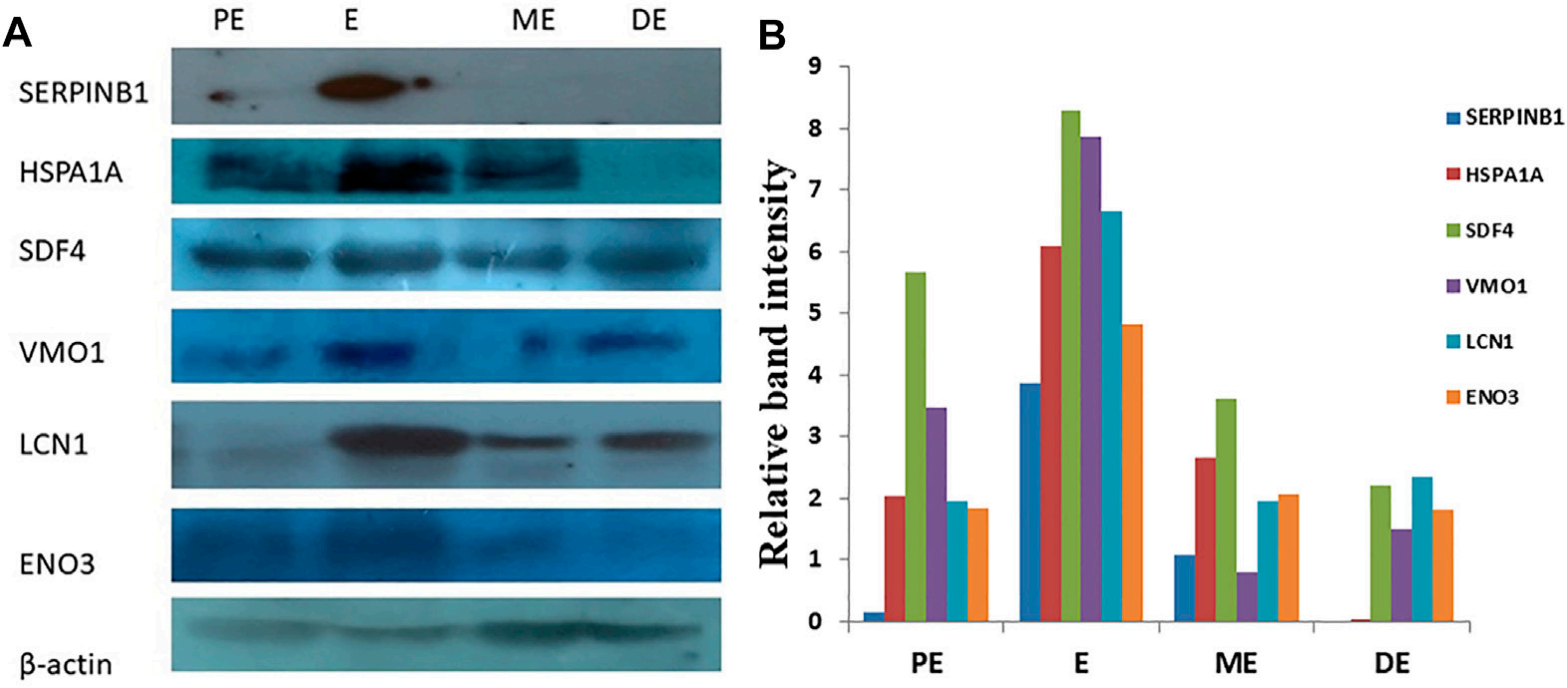
Western blot analysis for validation of differentially expressed upregulated proteins. **(A)** Over-expression of SERPINB1, HSPA1A, SDF4, VMO1, LCN1, and ENO3, during estrus (E) compared to proestrus (PE), metestrus (ME), and diestrus (DE) stages of the buffalo estrous cycle. **(B)** Band intensity was normalized against beta-actin as an internal control.

A total of six DEPs were selected for validation using Western blot analysis based on their involvement in different signaling pathways and their over-expression during E compared to all other non-estrus stages: SERPINB1, HSPA1A, SDF4, VMO1, ENO3, and LCN1. Interestingly, Western blot analysis confirmed the significantly higher expression of these proteins during E than PE, ME, and DE stages. The intensity of the protein expression was normalized with beta-actin as an internal control (Figure 11).

### 3.5 Gene Ontology Annotation of DEPs

**FIGURE 12:**
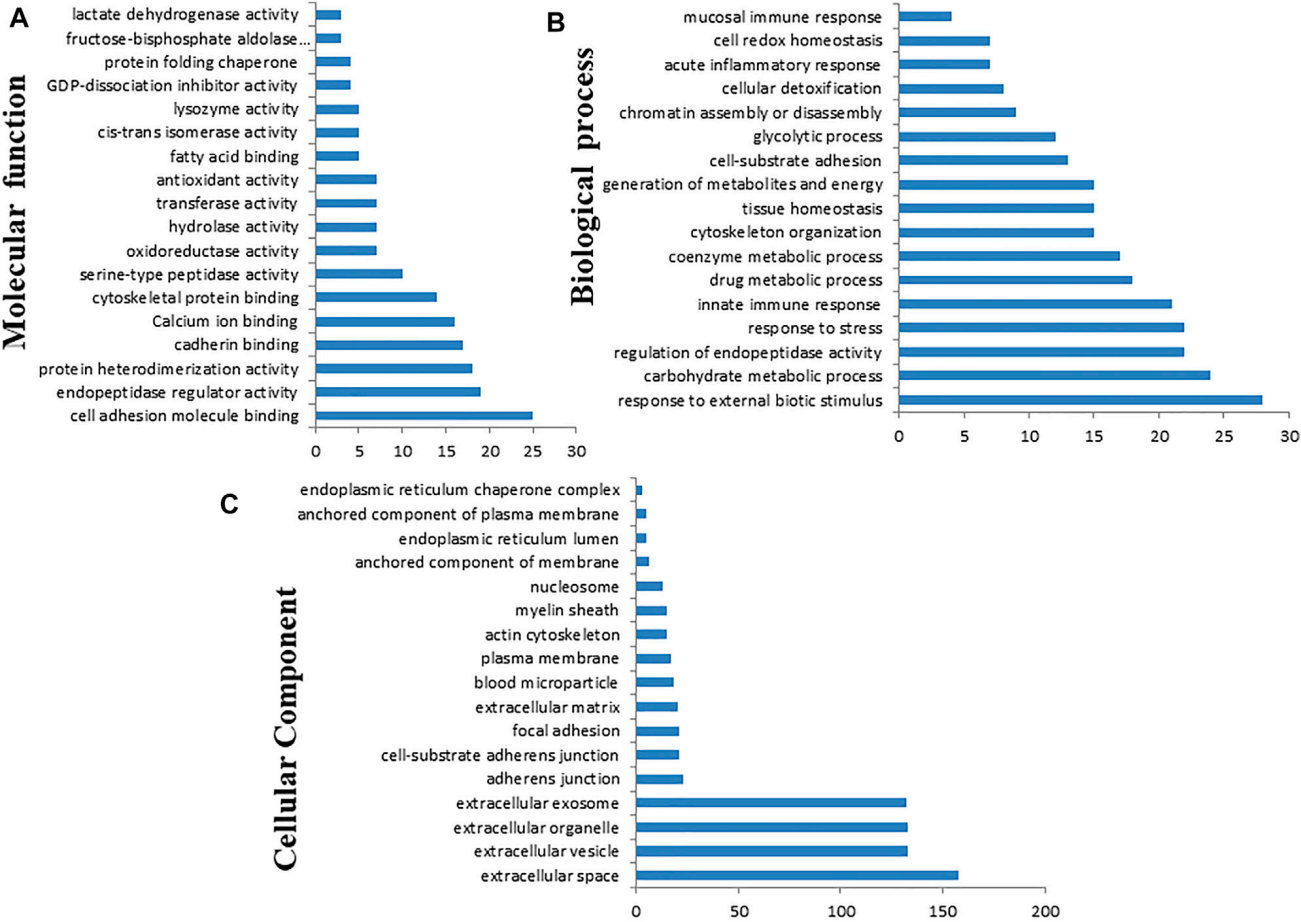
Gene Ontology (GO) classification of the differentially expressed proteins based on molecular function **(A)**, biological process **(B)**, and cellular component **(C)** using Cytoscape software with ClueGO plug-in.

Gene Ontology annotation of DEPs was performed on the Cytoscape ClueGo plug-in tool and presented in Figure 12. Based on the molecular function (Figure 12A, Supplementary Figure S1), the DEPs were found to be mainly enriched in cell adhesion molecule binding, endopeptidase regulator activity, protein heterodimerization activity, cadherin binding, cytoskeletal protein binding, oxidoreductase activity, hydrolase activity, transferase activity, fatty acid-binding, cis–trans isomerase activity, lysozyme activity, calcium ion binding, ion binding, antioxidant activity, and protein folding chaperone. Based on biological process (Figure 12B, Supplementary Figure S2), the DEPs were enriched in response to external biotic stimulus, metabolic process, regulation of endopeptidase activity, innate immune response, cytoskeleton organization, tissue homeostasis, generation of precursor metabolites and energy, ATP metabolic process, glycolytic process, chromatin assembly or disassembly, acute inflammatory response, cell redox homeostasis, and mucosal immune response. Based on the GO terms for cellular component (Figure 12C, Supplementary Figure S3), the DEPs belonged to endoplasmic reticulum lumen, plasma membrane, actin cytoskeleton, endoplasmic reticulum chaperone complex, myelin sheath, nucleosome, extracellular matrix, adherens junction, exosome, organelle, vesicle, and extracellular space.

### 3.6 Protein–Protein Interaction and Network Visualization

**FIGURE 13:**
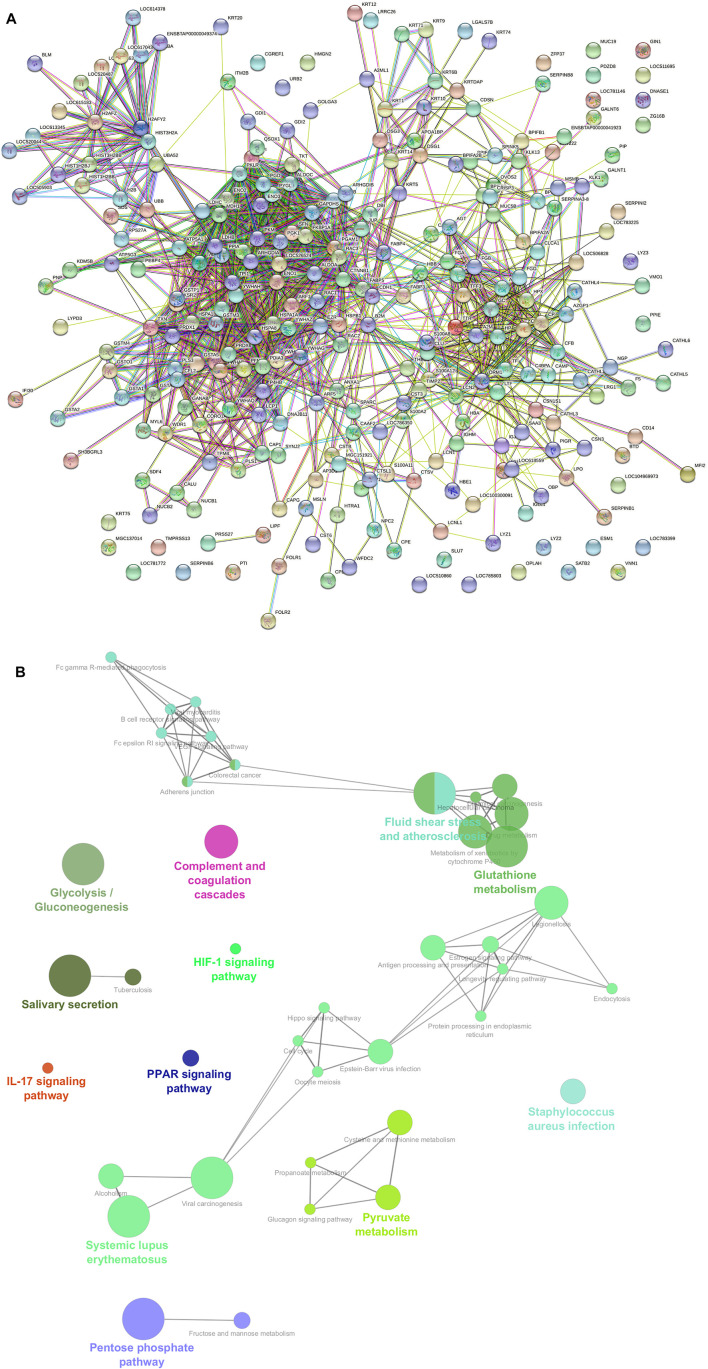
**(A)** Protein–protein interaction (PPI) network constructed by the STRING tool using combined score of 0.9–0.4. A PPI network with high combined score was created (*p* < 1.0E-16) with 378 nodes, 810 edges, 4.29 average node degree, and 0.357 average local clustering coefficient. **(B)** Functional annotation of a protein–protein interacting hub using Cytoscape software with ClueGO plug-in.

We performed protein–protein interaction (PPI) network analysis for DEPs using the STRING tool (v.11.5) with the *Bos taurus* database. The highly significant protein–protein interaction network (*p* < 1.0E-16) was created with 378 nodes, 810 edges, 4.29 average node degree, and 0.357 average local clustering coefficient (Figure 13A). A total of 31 clusters were found with the highest degree of connectivity among proteins. Furthermore, to explore the interactions between DEPs, we constructed protein networks using Cytoscape ClueGo plug-in tool (Figure 13B). A total 28 KEGG pathways were significantly enriched (*p* < 0.05), including glycolysis/gluconeogenesis, carbon metabolism, biosynthesis of amino acids, salivary secretion, fluid shear stress and atherosclerosis, glutathione metabolism, pentose phosphate pathway, innate immune response, antigen processing and presentation, oocyte meiosis, cell cycle, and estrogen signaling pathway (Table 6).

**TABLE 6:** Involvement of differentially expressed proteins (DEPs) in KEGG pathways.

S.No.	KEGG pathway	Number of genes	Associated gene	p-value
1	Glycolysis/gluconeogenesis	15	ALDOA, ALDOB, ALDOC, ENO1, ENO2, ENO3, GAPDHS, GPI, LDHA, LDHB, LDHC, PGAM1, PGK1, PKLR, and TPI1	0.00
2	PPAR signaling pathway	6	APOA1, DBI, FABP3, FABP4, FABP5, and UBC	0.00
3	Complement and coagulation cascades	9	A2M, C3, C4BPA, CFB, CLU, F5, FGA, FGB, and FGG	0.00
4	IL-17 signaling pathway	5	LCN2, LOC786350, MUC5AC, MUC5B, and S100A8	0.03
5	Salivary secretion	12	CAMP, CATHL2, CATHL3, CATHL4, CATHL5, CATHL6, CST3, LOC785803, LPO, LYZ2, LYZ3, and MUC5B	0.00
6	Pentose phosphate pathway	7	ALDOA, ALDOB, ALDOC, GPI, PGD, TALDO1, and TKT	0.00
7	Fructose and mannose metabolism	4	ALDOA, ALDOB, ALDOC, and TPI1	0.00
8	Cysteine and methionine metabolism	5	LDHA, LDHB, LDHC, MDH1, and SDS	0.00
9	Pyruvate metabolism	5	LDHA, LDHB, LDHC, MDH1, and PKLR	0.00
10	Propanoate metabolism	3	LDHA, LDHB, and LDHC	0.02
11	Glucagon signaling pathway	5	LDHA, LDHB, LDHC, PGAM1, and PYGL	0.03
12	VEGF signaling pathway	3	RAC1, RAC2, and RAC3	0.09
13	Adherens junction	5	CDH1, CTNNB1, RAC1, RAC2, and RAC3	0.01
14	B-cell receptor signaling pathway	4	LOC100300716, RAC1, RAC2, and RAC3	0.05
15	Fc epsilon RI signaling pathway	4	LOC100300716, RAC1, RAC2, and RAC3	0.04
16	Fc gamma R-mediated phagocytosis	5	CFL1, CFL2, LOC100300716, RAC1, and RAC2	0.02
17	Fluid shear stress and atherosclerosis	13	CTNNB1, CTSV, GSTA2, GSTA3, GSTA5, GSTM1, GSTM3, GSTO1, GSTP1, RAC1, RAC2, RAC3, and TXN	0.00
18	Glutathione metabolism	9	GSTA2, GSTA3, GSTA5, GSTM1, GSTM3, GSTO1, GSTP1, OPLAH, and PGD	0.00
19	Metabolism of xenobiotics by cytochrome P450	7	GSTA2, GSTA3, GSTA5, GSTM1, GSTM3, GSTO1, and GSTP1	0.00
20	Chemical carcinogenesis	7	GSTA2, GSTA3, GSTA5, GSTM1, GSTM3, GSTO1, and GSTP1	0.00
21	Fluid shear stress and atherosclerosis	13	CTNNB1, CTSV, GSTA2, GSTA3, GSTA5, GSTM1, GSTM3, GSTO1, GSTP1, RAC1, RAC2, RAC3, and TXN	0.00
22	Cell cycle	7	SFN, YWHAB, YWHAE, YWHAG, YWHAH, YWHAQ, and YWHAZ	0.01
23	Oocyte meiosis	6	YWHAB, YWHAE, YWHAG, YWHAH, YWHAQ, and YWHAZ	0.02
24	Protein processing in endoplasmic reticulum	8	GANAB, HSPA1A, HSPA1L, HSPA2, HSPA5, HSPA8, P4HB, and PDIA3	0.01
25	Endocytosis	11	ARF1, ARF3, ARF5, FOLR1, FOLR2, FOLR3, HSPA1A, HSPA1L, HSPA2, HSPA8, and UBB	0.01
26	Hippo signaling pathway	8	CDH1, CTNNB1, YWHAB, YWHAE, YWHAG, YWHAH, YWHAQ, and YWHAZ	0.01
27	Antigen processing and presentation	8	B2M, CTSV, HSPA1A, HSPA1L, HSPA2, HSPA8, IFI30, and PDIA3	0.00
28	Estrogen signaling pathway	4	HSPA1A, HSPA1L, HSPA2, and HSPA8	0.1

## 4 Discussion

Due to the difficulty of detecting estrus in buffalo, the finding of an estrus biomarker in readily accessible bodily fluids such as saliva is critical for the development of a simple, reliable estrus detection tool. Earlier investigation has confirmed the salivary proteins as a potential candidate to detect fertile periods in woman (Alagendran et al., 2013; Saibaba et al., 2021) and estrus (Muthukumar et al., 2014b; Shashikumar et al., 2018) in buffaloes. However, estrus biomarker discovery in buffalo saliva by differential proteome analysis using TMT and LFQ coupled to mass spectrometry is lacking. Comparative proteome profiling using LFQ-LC-MS/MS and TMT-LC-MS/MS analysis in buffalo saliva identified a total of 520 and 369 proteins as DEPs, respectively. These findings may be helpful for further understanding of buffalo estrus biology and development of an easy, reliable estrus detection method for buffalo.

### 4.1 Proteins Involved in Regulation of Endopeptidase Activity

Our study found the over-expression of leukocyte elastase inhibitor (SERPINB1) during estrus followed by proestrus and almost negligible expression during metestrus and diestrus stages of the estrous cycle. SERPINB1 and several other DEPs (A2M, A2ML1, C3, CST3, CST6, CSTB, LOC404103, LOC786263, LTF, OVOS2, PTI, S100A8, SERPINA3-8, SERPINB1, SERPINB6, SERPINB8, SERPINI2, SFN, SPINK5, THBS1, TIMP2, and WFDC2) in the present study were involved in the regulation of endopeptidase inhibitor activity. SERPINB1 and other protease inhibitors inactivate serine proteases and cysteine proteases and mediate important functions in fibrinolysis, coagulation, inflammation, cell mobility, cellular differentiation, apoptosis, and protein C pathways (Law et al., 2006), thus regulating the innate immune response, inflammation, and cellular homeostasis (Choi et al., 2019). The previous study demonstrated altered expression of three SERPINs, SERPINB2, SERPINE1, and SERPINE2 during follicular development in bovine ovarian follicles (Dow et al., 2002; Bedard et al., 2003). Expression of SERPINE2 gene in preovulatory follicles was markedly upregulated immediately after the LH surge and then decreased to the lowest level toward ovulation (Hasan et al., 2002; Cao et al., 2006). Similarly, over-expression of SERPINE2 in granulosa cells of mature follicles compared to that in small and medium follicles has also been observed (Cao et al., 2004). Previous studies also demonstrated over-expression of SERPINB6 in healthy follicles compared to that in the atretic ones, suggesting follicular development and atresia in bovine ovarian follicles are mediated through SERPINs (Hayashi et al., 2011). The increased abundance of SERPINB1 during the estrus stage may be a protective mechanism of follicular cells against several proteases and intrinsic for follicular development and final follicular maturation and steroid production by ovarian follicles.

### 4.2 Proteins Involved in Glycolysis and Pyruvate Metabolism

We observed increased expression of beta-enolase (ENO3) during E compared to PE, ME, and DE stages. ENO3 is a glycolytic enzyme and regulates the glycolysis process by catalyzing the reversible conversion of 2-phosphoglyceric acid to phosphoenolpyruvic acid (Giallongo et al., 1993). In addition, we observed 14 other DEPs (ALDOA, ALDOB, ALDOC, ENO1, ENO2, GAPDHS, GPI, LDHA, LDHB, LDHC, PGAM1, PGK1, PKLR, and TPI1) and five DEPs (LDHA, LDHB, LDHC, MDH1, and PKLR) were significantly involved in the glycolysis or gluconeogenesis metabolic pathway and pyruvate metabolism, respectively. It is speculated that these proteins associated with active synthesis of energy may support follicular development, steroidogenesis, final follicular maturation, and ovulation process. In a previous study, pyruvate metabolism has been essential for the completion of oogenesis, serving as a vital source of energy during the meiotic maturation of murine oocytes (Johnson et al., 2007). It has also been observed that the mature cumulus oocyte complex (COC) uses two times more glucose and pyruvate than the immature ones (Sutton et al., 2003), indicating high energy demand supported by upregulation of glycolysis by ovarian follicles during the process of final maturation of oocyte. The previous study has demonstrated that increased enolase expression is responsible for higher expression of the *FSH* gene in granulosa cells during the follicular phase (Hermann and Heckert, 2007). *FSH* has a significant role in reproduction by regulating granulosa cell differentiation, proliferation and ovarian steroidogenesis (Richards, 1994), and controlling follicular development and fertility in females (Hermann and Heckert, 2007). Specific expression of ENO3 in buffalo saliva, particularly during estrus stage has also been observed by an earlier study (Muthukumar et al. (2014b). The increased expression of ENO3 during E compared to other stages of the estrous cycle suggests it has a defined role in follicular development, final follicular maturation, and steroid production by follicular granulosa cells that leads to the onset of estrus in buffaloes.

### 4.3 Proteins Involved in Estrogen Signaling

This study also found a higher abundance of the heat shock protein family A (HSPA1A/HSP70) during estrus than other stages of the estrous cycle. HSPA1A is a molecular chaperon and plays a role in the estrogen signaling pathway. The present study also found other molecular chaperons such as HSPA1L, HSPA2, and HSPA8 as DEPs. Chaperones are stress-response molecules and involved in housekeeping of the cells and facilitating the transport, folding, unfolding, assembly, and disassembly of multi-structured protein units and degradation of misfolded or aggregated proteins (Sorensen et al., 2003). HSPA1A plays a role in the assembly and trafficking of steroid hormone receptors, acts as a co-activator for the nuclear estrogen receptor-α activity, and regulates steroid hormone synthesis by ovarian follicles (Khanna et al., 1995; Liu and Stocco, 1997; Hurd et al., 2000). HSPA1A also mediates luteal regression in murine corpus luteum (Khanna et al., 1995). A previous study also identified a higher abundance of HSPA1A protein in cervical mucus during E than the DE stage of the buffalo estrous cycle (Muthukumar et al., 2014a). Our previous study also observed estrus-specific expression of HSPA1A in buffalo saliva (Shashikumar et al., 2018). Furthermore, our direct saliva transcript analysis also demonstrated a higher level of HSP70 transcript in saliva during estrus than the diestrus stage, suggesting it as a good indicator of estrus in buffaloes (Onteru et al., 2016). HSPA1A protects cells from the negative effect of stress by promoting the folding of proteins and correcting the misfolding of denatured proteins (Lindquist and Craig 1988). Steroid hormone, particularly estradiol plays a key role in inducing estrus signs in farm animals, and its level increased from 22.4–35 pg/ml (Roy and Prakash 2009; Mondal et al., 2010) just before the onset of estrus in buffaloes. An earlier study demonstrated estradiol treatment-induced stress or injury in rat brain vasculature and estradiol-induced heat shock protein expression occurred as a protective mechanism to prevent cellular damage (Lu et al., 2002; Raval et al., 2013). Suggestive increased abundance of HSPA1A protein during estrus could be a protective mechanism of granulosa cells against estrogen-induced stress and its possible role in follicular development and steroidogenesis process.

### 4.4 Calcium Ion Binding Activity

Increased expression of 45-kDa calcium-binding protein (SDF4) was observed during E compared to PE, ME and DE stages of the estrous cycle. SDF4 is a calcium-binding protein and regulates calcium-dependent activities in the lumen of the endoplasmic reticulum and is involved in calcium-ion regulated exocytosis. Calcium-binding proteins are mainly localized in the cytosol and various parts of the secretory pathway (Honore, 2009). They have diversified functions, including secretory process, chaperone activity, and signal transduction (Honore, 2009). Although its exact role in estrous physiology is unknown, its higher expression during estrus suggests its possible involvement in chaperon-mediated steroidogenesis and cellular protection against estradiol-induced stress.

### 4.5 Proteins Involved in Innate Immune Response

The present study also identified several DEPs (PIGR, S100A12, BPIFA2B, B2M, A2M, BPIFB1, BPIFA1, CATHL3, CATHL5, FGB, and FGA) involved in innate immune response. The whole estrous cycle is regulated by the differential level of steroid hormones; estradiol is predominant during follicular phase and progesterone during the luteal phase. These steroid hormones modulate the immune system depending on the estrous cycle stage. Binding with specific hormone receptors, steroid hormone induces genomic and non-genomic actions in immune cells (Gilliver, 2010; Kovacs et al., 2010; Bellavance and Rivest, 2014). In general, estradiol is immune-enhancing by stimulating functions of innate immune cells, Th1 responses, and antibody production from B cells. However, progesterone is immune-suppressing by down-regulating functions of immune cells such as macrophages (Su et al., 2009), natural killer cells (Arruvito et al., 2008), or dendritic cells (Butts et al., 2007; Hughes et al., 2008). In addition, progesterone antagonizes the immune-enhancing function of estrogens on T-cell cycle progression (McMurray et al., 2001) and proliferation of T cells (Butts et al., 2007). Importance of antimicrobial peptides (S100A12, CATHL3, and CATHL5)(Diamond et al., 2009), BPI fold-containing family proteins (BPIFA2B, BPIFB1, and BPIFA1) (Li et al., 2020), polymeric immunoglobulin receptor (PIGR) (Wei and Wang, 2021), alpha-2-macroglobulin (A2M), and beta-2-macroglobulin (B2M) (Vandooren and Itoh, 2021) in mucosal immunity is well established. Mucosal immunity protects the internal genital tract against pathogens. During the estrus period, physical barriers between external genitalia and uterus breached, making pathogens to enter into the uterus. Hence, the estrogen-dominant estrus phase modulates the function of innate immune cells and secretes several antimicrobial peptides and proteins that protect the mucosa of the female reproductive tract against external pathogens.

### 4.6 Proteins Involved in Cytoskeleton Organization

The present study also depicted several DEPs involved in the cytoskeleton organization process (APOA1, ARF1, CAP1, CAPG, CFL1, CFL2, CORO1A, CSN1S1, EZR, FKBP1A, HSPA1A, KRT14, KRT20, KRT71, KRT9, LCP1, PFN1, PLS1, PLS3, RAC1, RAC2, and WDR1). It is during estrus that an oocyte that has been diplotene-restricted restarts meiosis before ovulation. Oocyte meiosis entails a number of intricate processes, including change of the mitotic cell cycle, chromosomal segregation, spindle reorganization, and accomplishment of oocyte asymmetry and polar body extrusion. These activities need reorganization of the cytoskeleton, coordination of cytoplasmic dynamics, and regulation of the cortex’s tension forces (Brunet and Verlhac, 2011; Yi et al., 2011). These findings and our results support the role of cytoskeletal proteins in follicular and oocyte maturation during estrus.

### 4.7 Proteins With Miscellaneous Functions

Notably, we noticed an elevated expression of vitelline membrane outer layer protein 1 (VMO1) in comparisons between E and PE, ME, and DE. It is the first report of VMO1 protein in saliva during the estrous cycle. Its precise involvement in controlling folliculogenesis and estrous physiology is unknown. VMO1 protein was initially found in chicken as an important component of the egg’s vitelline membrane’s outer layer (Kido et al., 1992) and plays a critical function in oviductal recrudescence in laying hens (Lim and Song, 2015). It is anticipated to be a new estrogen-induced gene in laying hens and a possible diagnostic biomarker for ovarian cancer (Lim and Song, 2015). As a result, it is probable that the estradiol peak that occurs during estrus is the cause for the over-expression of VMO1 and its significant relationship with estrus physiology. Additional investigations in large animals using powerful genetic engineering technologies such as CRISPR-based knockout are required to characterize the mechanism of action (Singh et al., 2000). Another protein, nucleobindin-1 (NUCB1), was shown to be significantly increased during estrus compared to other non-estrus phases. Nucleobindins are multidomain Ca2+ and DNA-binding proteins that have a variety of roles in vertebrates. NUCB1 is converted from NUCB2 to biologically active NUCB1 by the action of prohormone convertase (Rajeswari et al., 2020). NUCB2 is claimed to operate as a local regulator in the mouse ovary, controlling steroidogenesis and energy balance through autocrine and paracrine signaling (Kim and Yang, 2012). Additionally, NUCB2 has been shown to bind predominantly to epithelial cells of uterine glands and neutrophils of the endometrium during the mouse estrous cycle, suggesting that its expression in the uterus may be regulated by estrogen secreted by the ovary but not by gonadotropin released by the pituitary gland (Kim et al., 2011). The precise involvement of NUCB1 in estrous physiology in farm animals is unknown and requires additional research. Additionally, the current research showed that two proteins, lipocalin 1 (LCN1) and odorant-binding protein-2B (OBP2B), were overexpressed during estrus compared to other non-estrus phases. Lipocalin one is a globular protein that interacts with and transports a variety of ligands including volatile pheromones/odorants, fatty acids, lipids, steroids, bilins, and retinol. Lipocalin has been extensively studied as a chemosignal in a variety of animals, including rats and pigs (Scaloni et al., 2001; Muthukumar et al., 2013; Cerna et al., 2017). It was more abundant in rodent urine during estrus than during diestrus (Muthukumar et al., 2013; Cerna et al., 2017). Additionally, it was isolated from bovine olfactory mucosa (Pevsner et al., 1986). Lipocalin is connected with chemical communication during estrus, that is, a unique volatile signal sent by urine from a female to male partner as a mating call (Brennan and Keverne, 2004). Lipocalin has also been identified as a significant progesterone-dependent protein released into the uterine lumen in mares (Crossett et al., 1996). Similarly, over-expression of odorant-binding protein during estrus compared to other stages of the estrous cycle was observed. Odorant-binding protein (OBP) plays a role in odor perception, olfactory stimulus, and chemical communication, especially in insects and mammals through body fluids (Pelosi and Maida, 1990). Cerna et al. (2017) also identified OBP as a pheromone-binding protein in the vaginal fluid of mice and observed its highest concentration before ovulation. OBP is an essential protein for the communication signal in rodents (Cavaggioni and Mucignat-Caretta, 2000; Rajkumar et al., 2009). A previous study also identified this protein in buffalo saliva ((Rajkumar et al., 2010; Muthukumar et al., 2018). Increased expression of LCN1 and OBP2B during estrus compared to other non-estrus phases implies that they play a critical role in mediating chemosignaling during estrus in buffalo.

## Conclusion

This is the first comprehensive report on differential proteome analysis of buffalo saliva between the stages of estrus and non-estrus by employing both label-free and TMT-based labeled quantitation and mass spectrometry analysis. Numerous proteins involved in endopeptidase activity, glycolysis, pyruvate metabolism, calcium ion binding, estrogen signaling, and pheromone signaling were identified as differentially expressed between estrus and non-estrus phases. The extensive saliva proteome data set obtained in this work will provide insights into the cellular processes behind buffalo estrous biology. Taken together, this work identified a critical panel of potential proteins in saliva that may be used as a biomarker for buffalo estrus and paves the way for the development of a diagnostic kit for buffalo estrus detection.

## Data Availability

The datasets presented in this study can be found in online repositories. The names of the repository/repositories and accession number(s) can be found in the article/Supplementary Material.
