# Supplementary figures and images for: Comparative Proteome Profiling of Saliva Between Estrus and Non-Estrus Stages by Employing Label-Free Quantitation (LFQ) and Tandem Mass Tag (TMT)-LC-MS/MS Analysis: An Approach for Estrus Biomarker Identification in Bubalus bubalis
Source: Front Genet. 2022 May 13;13:867909. doi: 10.3389/fgene.2022.867909 (PMC9217162; doi:10.3389/fgene.2022.867909)

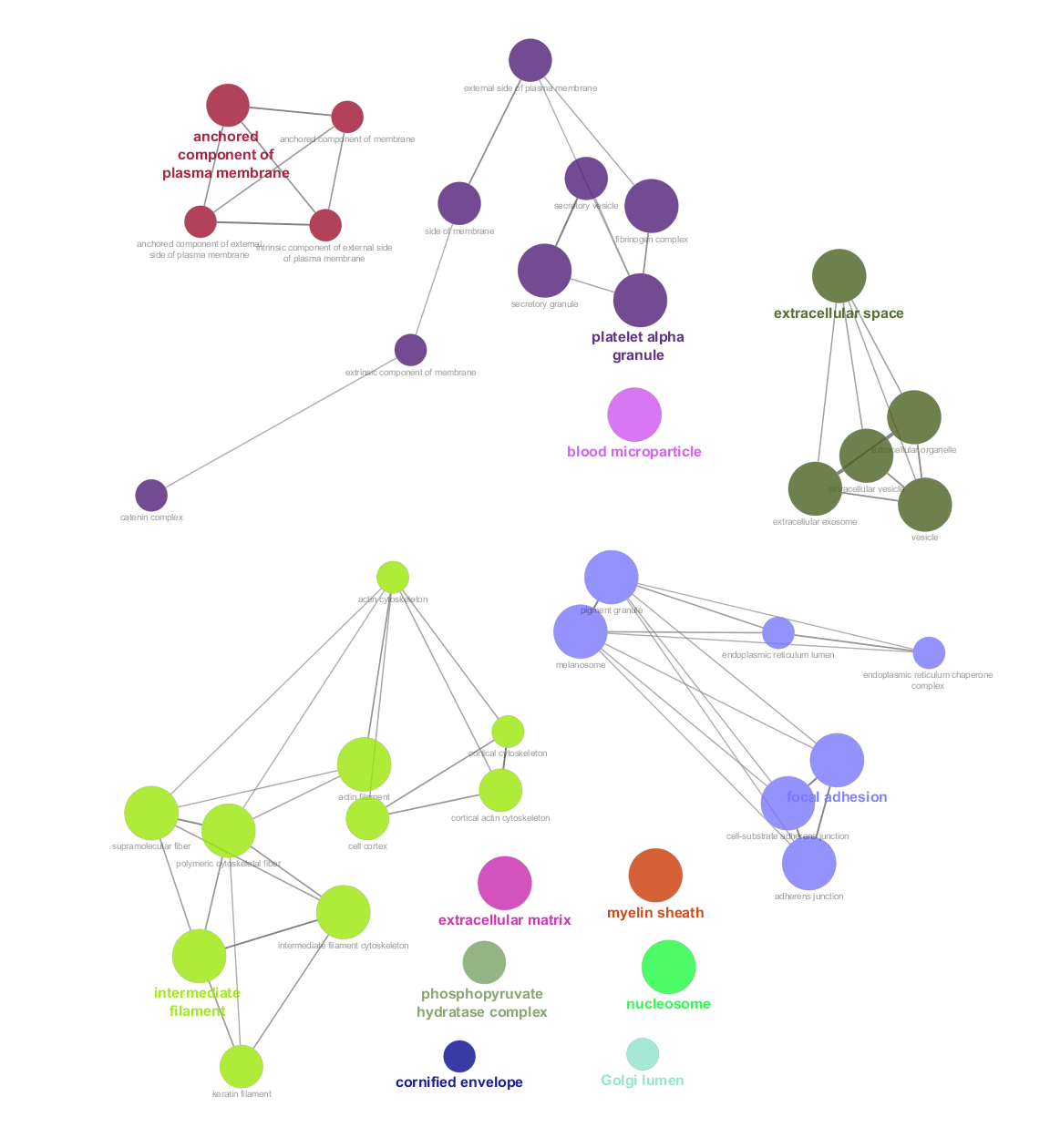

Supplement: Supplementary file 2 [file Image3.TIF]

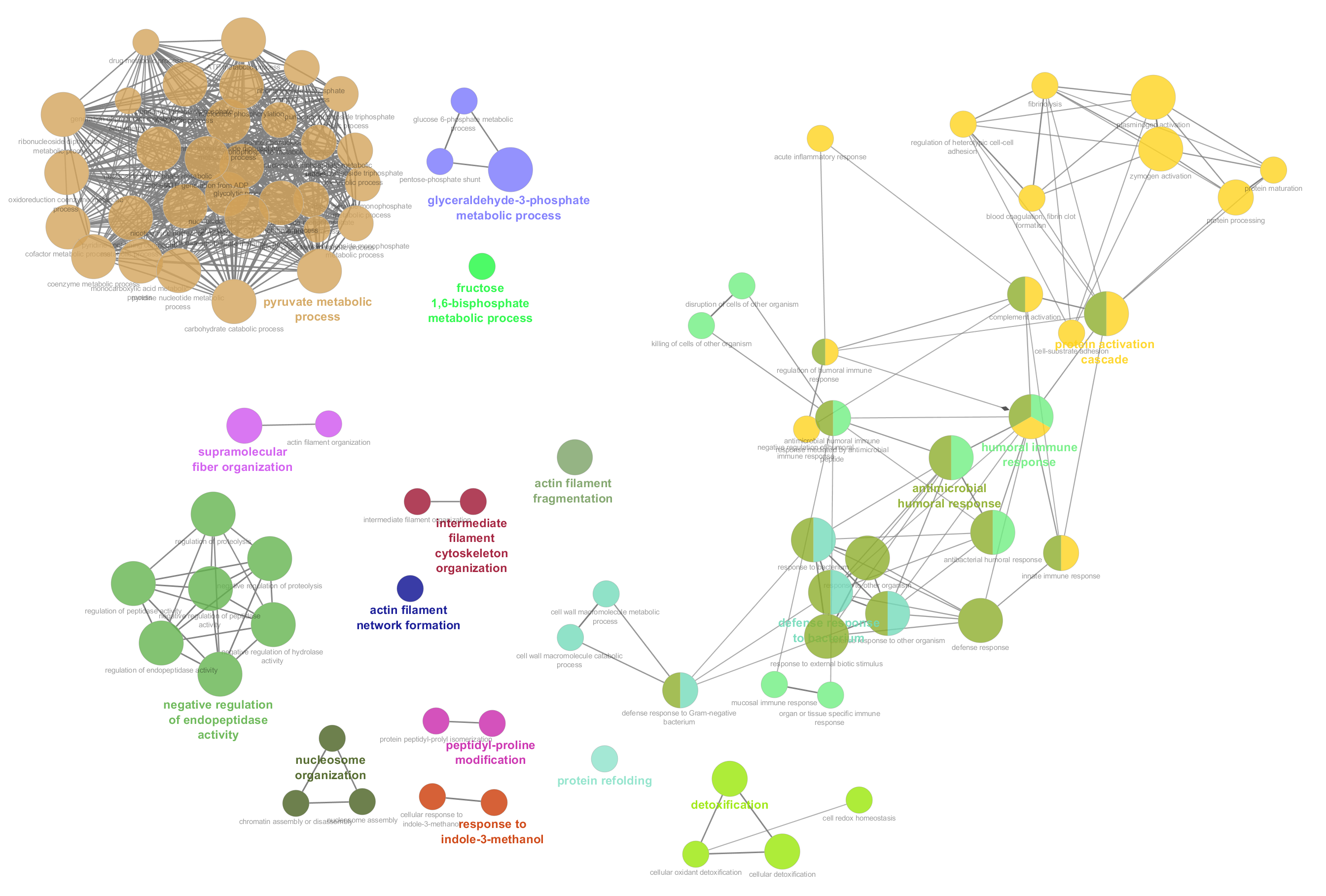

Supplement: Supplementary file 3 [file Image2.TIF]

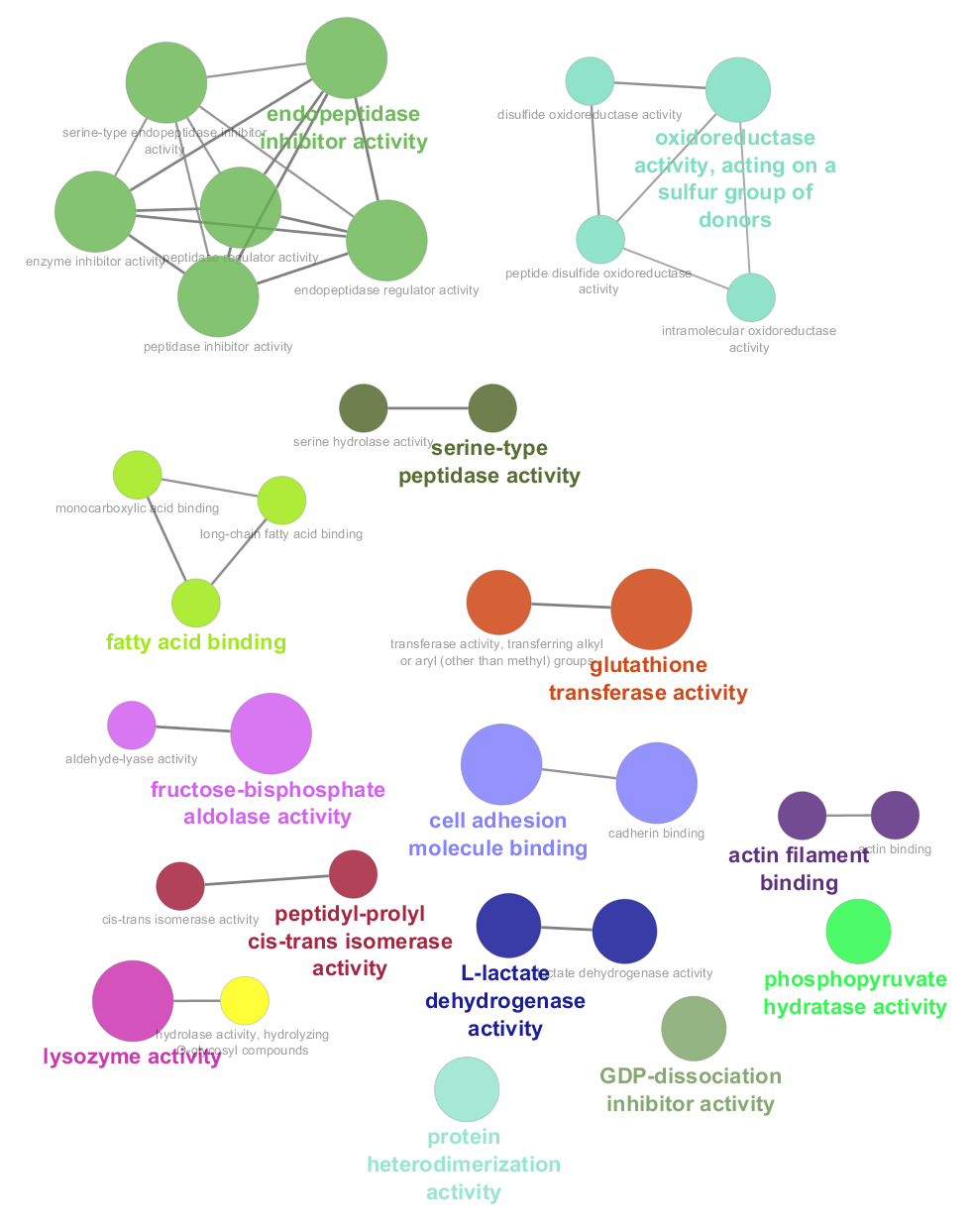

Supplement: Supplementary file 4 [file Image1.TIF]
